# PGC-1α inhibits M2 macrophage polarization and alleviates liver fibrosis following hepatic ischemia reperfusion injury

**DOI:** 10.1038/s41420-023-01636-2

**Published:** 2023-09-07

**Authors:** Yanting Zhang, Linzhong Zhang, Yanmian Zhao, Jing He, Yanghao Zhang, Xiuying Zhang

**Affiliations:** 1 Department of Histology and Embryology, School of Basic Medical Sciences, Capital Medical University, Beijing, China; 2 Department of Gastroenterology, Air Force Medical Center, Beijing, China; 3 Department of Internal Medicine, School Hospital, Communication University of China, Beijing, China

**Keywords:** Liver fibrosis, Experimental models of disease

## Abstract

Oxidative stress can induce inflammation, promoting macrophage polarization and liver fibrosis following hepatic ischemia-reperfusion (I/R). Peroxisome proliferator-activated receptor-γ coactivator-1α (PGC-1α) has anti-oxidant and anti-inflammatory activity. However, how PGC-1α regulates macrophage polarization following hepatic I/R remains largely unknown. Male C57BL/6 wild-type mice were pre-treated with vehicle or trichostatin A (TSA) for 2 days and subjected to surgical induction of I/R. Liver injury and fibrosis in individual mice were examined longitudinally and the expression levels of IL-6, STAT3, M2-type macrophage markers, Collagen I and α-SMA in the liver of mice were analyzed by immunohistochemistry, RT-qPCR and Western blot. The potential interaction of PGC-1α with phosphorylated NF-kBp65 was determined by immunoprecipitation. The impacts of PGC-1α deficiency in hepatocytes on their IL-6 production and macrophage polarization were tested in a Transwell co-culture system. Moreover, the M2-type macrophage polarization and liver fibrosis were examined in hepatocyte-specific PGC-1α knockout mice and AAV8-mediated PGC-1α over-expressing mice following liver I/R. The down-regulated PGC-1α expression by I/R was negatively correlated with IL-6 levels in the liver of I/R mice and PGC-1α deficiency enhanced IL-6 expression, STAT3 activation and M2-type macrophage polarization in the I/R mice, which were abrogated by TSA treatment. In addition, PGC-1α directly interacted with phosphorylated NF-kBp65 in I/R livers. Hepatocyte-specific PGC-1α deficiency increased IL-6 production and promoted macrophage polarization toward M2 type when co-culture. More importantly, administration with AAV8-PGC-1α rescued the I/R-induced liver fibrosis by inhibiting the IL-6/JAK2/STAT3 signaling and M2-type macrophage polarization in the liver. These results suggest that PGC-1α may alleviate the I/R-induced liver fibrosis by attenuating the IL-6/JAK2/STAT3 signaling to limit M2-type macrophage polarization. PGC-1α may be a therapeutic target for the treatment of liver fibrosis.

## Introduction

Hepatic ischemia reperfusion injury (IRI) is a common complication of post-liver surgery that is involved in hepatic devascularization, such as hemorrhagic shock, liver transplantation, and others [1, 2]. IR can induce innate immune response in the liver by activating macrophages that produce many cytokines and chemokines, leading to activation of hepatic stellate cells (HSCs). Subsequently, the activated HSCs, together with collagen fiber deposition in extracellular matrix (ECM), contributing to the process of hepatic fibrosis [3–5]. A previous study reported that M1-polarized macrophages alleviated the severity of liver fibrosis in mice [6]. However, the precise role of macrophages in the process of liver fibrosis following I/R has not been clarified.

Macrophages are important innate immune cells and naïve macrophages can be classically activated by lipopolysaccharides (LPS), INF-γ and interleukin (IL)-12 to be M1-type cells. Pro-inflammatory M1-type macrophages express CD80, CD86, IL-1R, inducible nitric oxide synthase (iNOS) and secrete tumor necrosis factor-α (TNF-α), IL-1β, IL-6 and some chemokines. Functionally, the M1-type macrophages are critical for defensing against bacterial and virus infection and promoting inflammation. Furthermore, macrophages can also be alternatively activated to become M2-type cells, which express CD204, CCR2, and chitinase 3-like 3 (Ym-1). M2-type macrophages produce anti-inflammatory IL-10, transforming growth factor (TGF)-β, IL-4, IL-13, and others, inhibiting inflammation and participating in tissue remodeling [7, 8]. These two types of macrophages have the different or even opposite functions during the process of different diseases and their functional balance determines homeostasis in the body [9, 10]. In addition, macrophage phenotype transformation can also contribute to the process of a variety of diseases, such as Salmonella typhimurium infection, necrotic colitis, hepatitis C infection, systemic lupus erythematosus and brain injury [7, 10–12]. Nevertheless, whether there is phenotypic transformation, what the function of liver macrophages is during the process of I/R-induced liver fibrosis, and what factors regulate this process have not been well elucidated. Therefore, characterizing the mechanisms underlying macrophage polarization during the process of I/R-induced liver fibrosis may help in discovering new therapeutic targets.

Trichostatin A (TSA), a histone deacetylase inhibitor, is the most commonly used drug to study the effect of protein acetylation on gene expression [13, 14]. PGC-1α is a transcriptional coactivator of nuclear receptors, and important in maintaining cellular energy metabolism, oxidative stress response, and immune inflammation. PGC-1α expression is closely related to the inflammatory process, and exogenous PGC-1α can restore kidney injury caused by systemic inflammation [15]. However, it is not clear whether PGC-1α can regulate macrophage polarization and the process of liver fibrosis following hepatic I/R injury.

A previous study has shown that liver fibrosis is reversible under certain conditions [16]. It is of great significance to prevent hepatic I/R progression to liver fibrosis. Our previous work has shown that oxidative stress can inhibit PGC-1α transcription by promoting histone deacetylation at the promotor zone. Accordingly, we hypothesize that hepatocyte PGC-1α deficiency caused by oxidative stress following I/R may regulate macrophage polarization by modulating the IL-6/JAK2/STAT3 signaling. Hence, administration with exogenous AAV8-PGC-1α to induce PGC-1α over-expression in the liver may be an effective strategy for the treatment of liver fibrosis following hepatic I/R injury.

## Results

### PGC-1α expression is significantly downregulated in the liver of mice following hepatic I/R injury and fibrosis

**Fig. 1:**
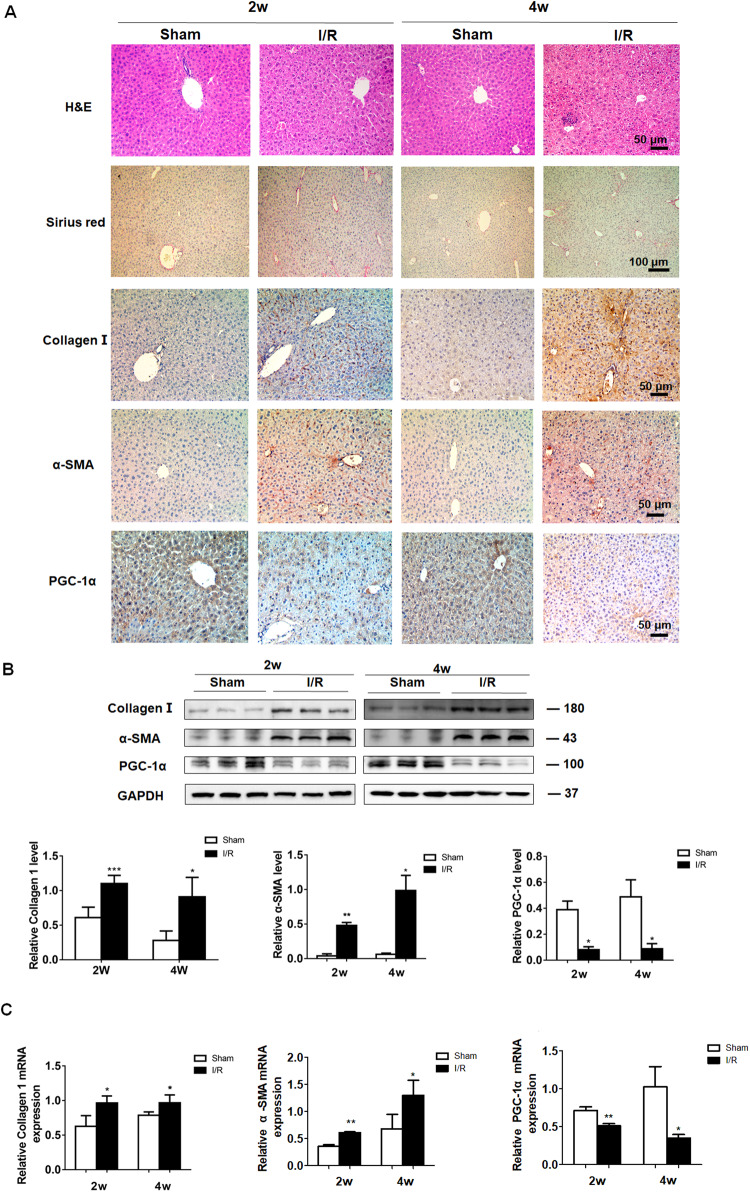
PGC-1α expression is significantly down-regulated in fibrotic liver of I/R mice. **A** H&E, Sirus red staining and immunohistochemistry analyses of Collagen I, α-SMA and PGC-1α expression in liver tissues of mice at 2 and 4 weeks post I/R. **B**, **C** Western blot and RT-qPCR analyses of the relative levels of Collagen I, α-SMA and PGC-1α expression in liver tissues of mice at 2 and 4 weeks post I/R. Data are representative images or shown as mean ± SD of each group (6–10/group) from at least three separate experiments. *P < 0.05, **P < 0.01, ***P < 0.001.

The presence and severity of liver fibrosis were examined in mice at 2 and 4 weeks post hepatic I/R injury by H&E and Sirius red staining. Compared with the mice in the Sham group, there were obviously hepatic fibrosis, inflammation and some degrees of necrosis in the liver of mice at 2 and 4 weeks following I/R induction, and immunohistochemistry exhibited remarkably increased Collagen I and α-SMA expression (Fig. 1A). In contrast, obviously down-regulated PGC-1α expression was observed in the liver of mice in the I/R group. Similar patterns of Collagen I, α-SMA and PGC-1α expression were detected in different groups of mice by Western blot and RT-qPCR (Fig. 1B, C). Hence, significantly down-regulated PGC-1α expression was associated with the progression of liver fibrosis in mice following induction of I/R injury.

### Decreased PGC-1α expression is paralleled with increased oxidative stress in the liver of mice at the early stage of hepatic I/R injury

**Fig. 2:**
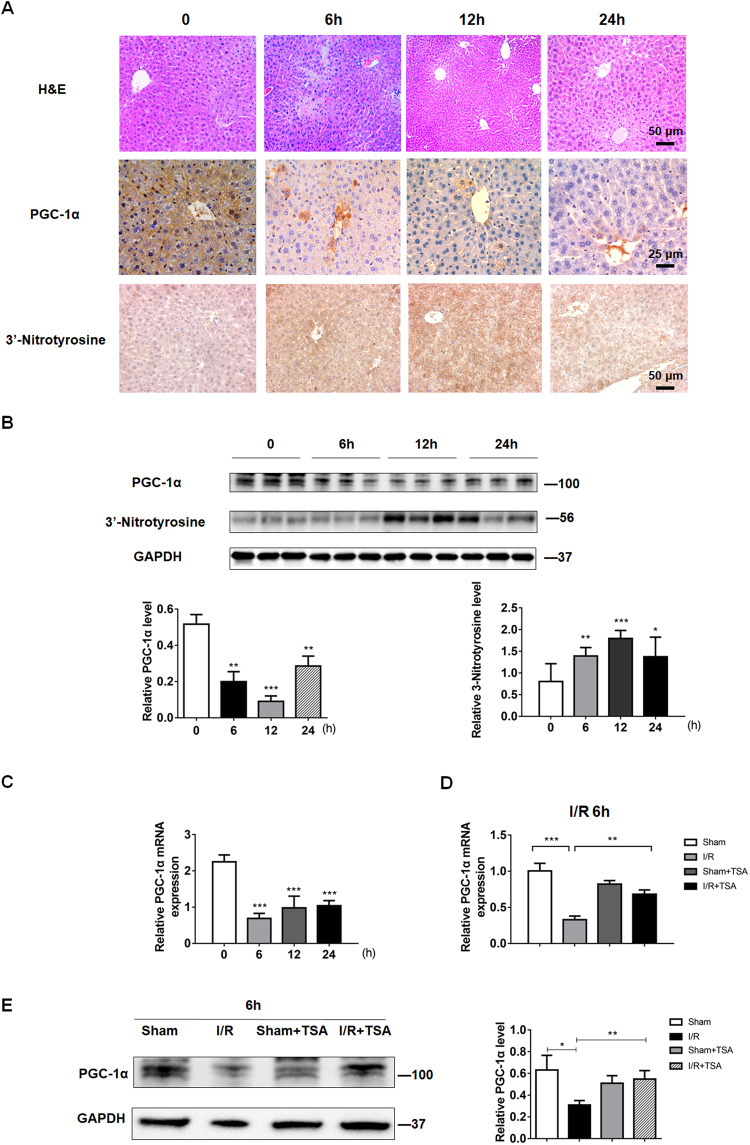
Down-regulated PGC-1α expression is associated with increased oxidative stress in the liver of mice at the early stage of hepatic I/R injury. **A** H&E and immunohistochemistry analyses of 3’-nitrotyrosine and PGC-1α expression in liver tissues of mice at 0, 6, 12 and 24 h post hepatic I/R. **B**, **C** Western blot and RT-qPCR analyses of the relative levels 3’-nitrotyrosine and PGC-1α protein expression (**B**), and PGC-1α mRNA transcripts in liver tissues of mice at indicated time points post hepatic I/R (**C**), respectively. **D**, **E** TSA treatment partially rescued PGC-1α mRNA (**D**) and protein (**E**) levels in liver tissues of mice at 6 h post hepatic I/R. Data are representative images or shown as mean ± SD of each group (6–10/group) from at least three separate experiments. *P < 0.05, **P < 0.01, ***P < 0.001.

PGC-1α is a coactivator of peroxisome proliferator-activated receptor γ (PPARγ), which is crucial for regulating glucose and lipid metabolism and inflammatory response [17, 18]. Given that I/R induces oxidative stress, we explored the relationship between PGC-1α expression and oxidative stress. As shown in Fig. 2A, obviously up-regulated expression of 3-nitrotyrosine (a biomarker of oxidative damage) paralleled with down-regulated PGC-1α expression in liver tissues of mice at 6, 12, and 24 h post hepatic I/R injury. Similar results were obtained by Western blot (Fig. 2B). Our previous work has shown that oxidative stress decreases PGC-1α expression by inhibiting histone acetylation at the promoter zone [19]. RT-qPCR revealed that PGC-1α mRNA transcripts decreased in the liver of mice at 6, 12, and 24 h following hepatic I/R injury, particularly at 6 h post hepatic I/R injury (Fig. 2C). In contrast, treatment with TSA partially rescued the levels of PGC-1α mRNA transcription (Fig. 2D), and protein expression (Fig. 2E) in the liver of mice. Thus, hepatic I/R-related oxidative stress limited PGC-1α expression in the liver of mice at the early stage of I/R-related injury.

### TSA treatment preserves PGC-1α expression and attenuates liver injury in mice at early stage of I/R-related injury

**Fig. 3:**
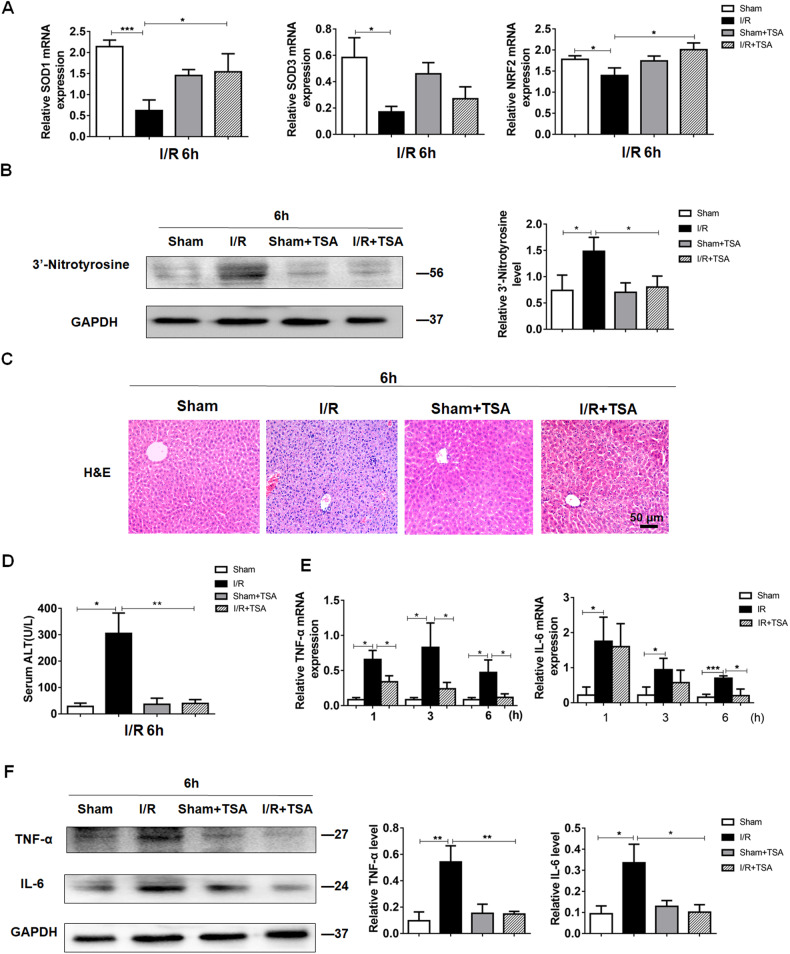
TSA treatment preserves PGC-1α expression to mitigate oxidative stress, inflammation and liver injury in mice at early time post I/R. Liver tissues were harvested from the indicated groups of mice at 6 h post hepatic I/R. **A** RT-qPCR analyses of the relative levels of SOD1, SOD3 and NRF2 mRNA transcripts. **B**, **F** Western blot analyses of the relative levels of 3’-nitrotyrosine (**B**), TNF-α and IL-6 (**F**) protein expression in liver tissues of mice. **C** H&E staining. **D** The levels of serum ALT. **E** RT-qPCR analysis of the relative levels of TNF-α and IL-6 mRNA transcripts in liver tissues from the indicated groups of mice at 1, 3 and 6 h post hepatic I/R. Data are representative images or shown as mean ± SD of each group (6–10/group) from at least three separate experiments. *P < 0.05, **P < 0.01, ***P < 0.001.

Given that PGC-1α can inhibit oxidative stress [20, 21], we tested the impact of TSA treatment on the expression of superoxide dismutase (SOD)1, SOD3 and NF-E2–related factor 2 (NRF2) in the liver of mice by RT-qPCR. The results indicated that compared with the I/R group, treatment with TSA significantly mitigated the I/R-decreased SOD1, and NRF2, but not SOD3, expression in the liver of mice at 6 h post I/R induction (Fig. 3A). Conversely, TSA treatment prevented the I/R-increased 3’-nitrotyrosine expression and attenuated liver injury in the liver of mice at 6 h post I/R induction, accompanied by limiting the I/R-increased serum ALT levels (Fig. 3B–D). Next, we questioned whether TSA treatment could alleviate the expression of inflammatory factors at the early stage of I/R-injury in mice. RT-qPCR revealed that compared with the Sham group, I/R increased the expression of TNF-α and IL-6 in the liver of mice at 1, 3 and 6 h post I/R induction while TSA treatment alleviated the I/R-increased TNF-α and IL-6 expression (Fig. 3E, F). Accordingly, the preserved PGC-1α expression by TSA treatment alleviated liver damage and inflammation in mice.

### TSA treatment preserves PGC-1α expression and suppresses the process of liver fibrosis in mice

**Fig. 4:**
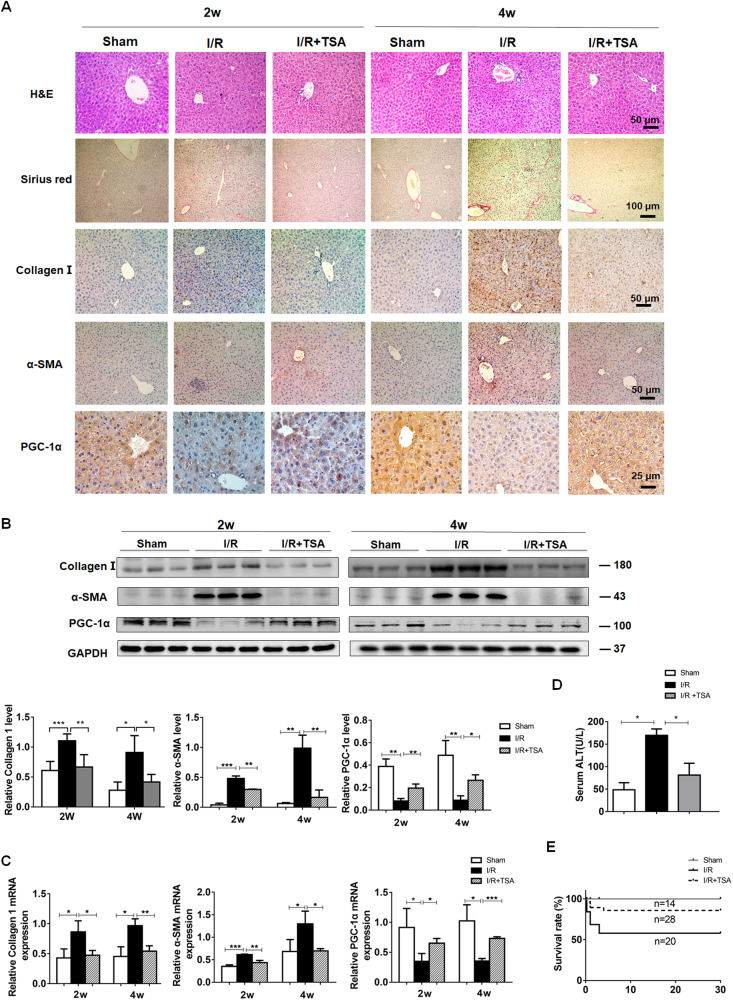
TSA treatment preserves PGC-1α expression and suppresses liver fibrosis in I/R mice. Two and four weeks after hepatic I/R, liver tissues were collected from the indicated groups of mice. The levels of liver injury, fibrosis and Collagen I, α-SMA and PGC-1α expression in individual liver samples were analyzed by H&E, Sirus red staining and immunohistochemistry (**A**), Western blot (**B**) and RT-qPCR (**C**). **D** The levels of serum ALT in individual mice. **E** The survival rates of each group of mice, measured by the Kaplan-Meier method. Data are representative images or shown as mean ± SD of each group (14–28/group) from at least three separate experiments. *P < 0.05, **P < 0.01, ***P < 0.001.

Inflammation can drive the process of liver fibrogenesis [22], but the molecular mechanisms by which PGC-1α regulates liver fibrosis caused by I/R are unclear. Compared to I/R mice, TSA treatment partially rescued the I/R-decreased PGC-1α expression in liver tissues of mice at 2 and 4 weeks post hepatic I/R injury (Fig. 4A). The TSA-treated I/R mice displayed less hepatic injury, lower serum ALT levels and fibrosis (Fig. 4A, D). Consistently, Western Blot and RT-qPCR revealed that TSA treatment mitigated the I/R-increased Collagen I and α-SMA expression in liver tissues of mice (Fig. 4B, C). Importantly, while I/R reduced the survival rate of mice, treatment with TSA only slightly increased the survival rate of mice, relative to that in the Sham group (Fig. 4E). Collectively, TSA treatment preserved PGC-1α expression to mitigate the I/R-induced liver fibrosis in mice.

### PGC-1α inhibits IL-6 expression by attenuating the NF-kBp65 signaling in the livers of mice following I/R induction

**Fig. 5:**
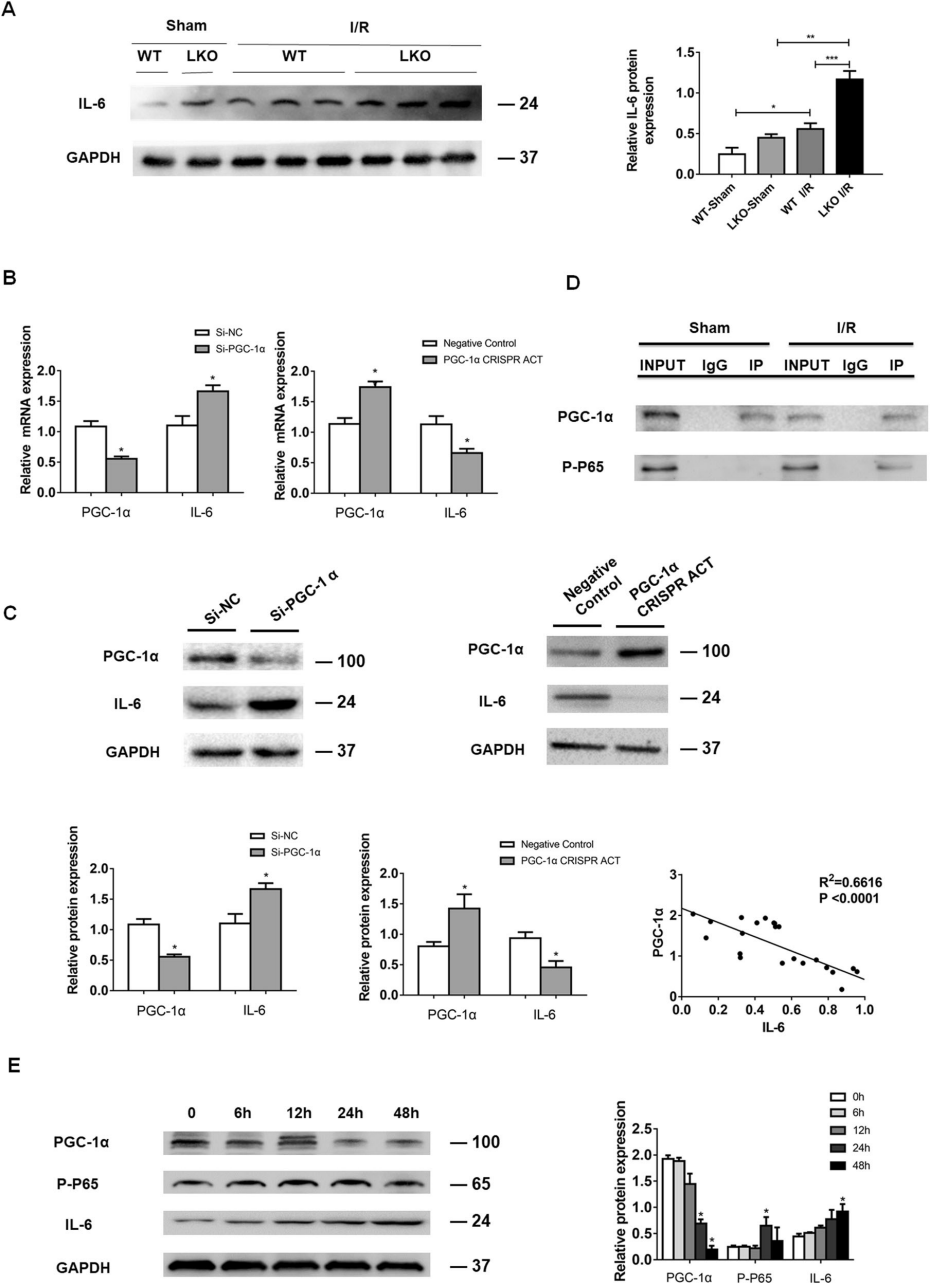
PGC-1α deficiency promotes IL-6 expression. **A** Western blot analyses of the relative levels of hepatic IL-6 protein expression in PGC-1α^f/f^albcre^+/0^ mice and wild-type mice at 2 weeks post I/R. **B**, **C** Altered PGC-1α expression modulated IL-6 expression in hepatocytes. Primary mouse hepatocytes were transfected with control si-NC or PGC-1α-specific siRNA (si-PGC-1α), control or PGC-1α CRISPR activation plasmid (PGC-1α CRISPR ACT) and the levels of PGC-1α and IL-6 mRNA transcripts and protein expression were tested by RT-qPCR (**B**) and Western blot as well as their negative correlation (**C**). **D** Immunoprecipitation analysis of the direct interaction of PGC-1α with (Ser536) phosphorylated NF-kBp65 in liver tissues of mice at 2 weeks post I/R using IgG as a control. **E** Western blot analysis of the relative levels of PGC-1α and IL-6 expression and NF-kBp65 phosphorylation in L-02 cells after treatment with 300 μM H_2_O_2_ for up to 48 h. Data are representative images or shown as mean ± SD of each group (6–8/group) from at least four separate experiments. *P < 0.05, **P < 0.01, ***P < 0.001.

IL-6 through its receptors of IL-6R (CD236) and glycoprotein 130 can activate the Janus kinases (JAK) and signal transducer and activator of transcription 3 (STAT3) signaling, enhancing liver fibrosis [23, 24]. PGC-1α deficiency can up-regulate IL-6 expression by enhancing the NF-kB signaling during the process of acute pancreatitis [25]. However, it is still unclear how PGC-1α influences IL-6 expression during the process of liver fibrosis induced by hepatic I/R injury. Western blot revealed that compared with the Sham group, hepatocyte-specific PGC-1α knockout (LKO) mice displayed significantly higher levels of IL-6 expression in the liver at 2 weeks post I/R induction (Fig. 5A). Similarly, while PGC-1α silencing by siRNA technology significantly increased IL-6 expression, PGC-1α over-expression decreased IL-6 expression in primary hepatocytes (Fig. 5B, C). As a result, the levels of PGC-1α expression were negatively correlated with IL-6 levels in the liver of mice following I/R induction (*p* < 0.001). Furthermore, immunoprecipitation unveiled that anti-PGC-1α rarely precipitated phosphorylated NF-kBp65 in the liver of mice from the Sham group, but effectively captured phosphorylated NF-kBp65 in the liver of I/R mice at 2 weeks post I/R induction (Fig. 5D). Consistently, Western blot indicated that PGC-1α expression gradually decreased, while NF-kBp65 phosphorylation and IL-6 expression progressively increased in the H_2_O_2_-treated primary hepatocytes in a time-dependent manner (Fig. 5E). Together, these data indicated that PGC-1α inhibited IL-6 expression by directly interacting with phosphorylated NF-kBp65 in the liver of I/R mice.

### PGC-1α deficiency enhances M2-type macrophage polarization through the IL-6/STAT3 signaling in the liver of mice

**Fig. 6:**
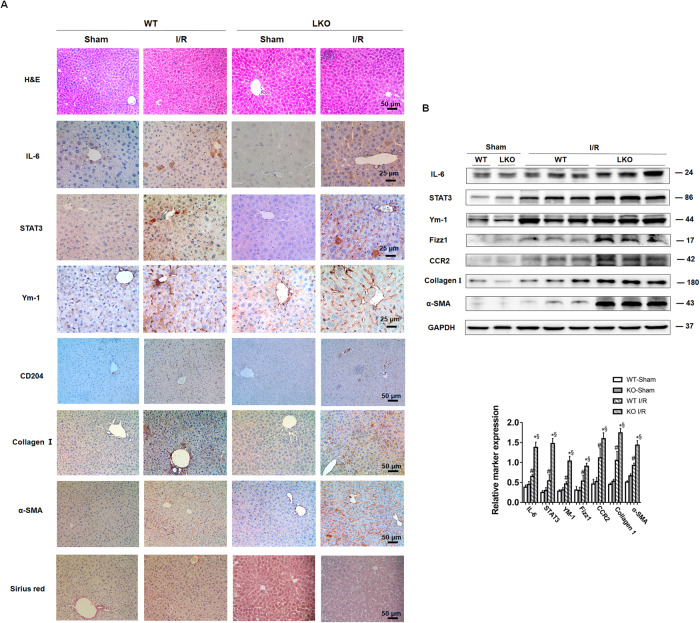
PGC-1α deficiency enhances the IL-6/STAT3 signaling and M2-type macrophage polarization in the liver of mice. Two weeks after I/R, the levels of liver injury and fibrosis as well as the indicated marker expression in the liver of PGC-1α^f/f^albcre^+/0^ (LKO) and wild-type mice were analyzed by H&E, Sirius red and immunohistochemistry staining (**A**) as well as Western blot (**B**). Data are representative images or shown as mean ± SD of each group (4–8/group) from at least four separate experiments. ^#^*P* < 0.01 vs. the WT-Sham, *P < 0.01 vs. the LKO-Sham, ^§^*P* < 0.01 vs. the WT-I/R.

IL-6 can regulate M2-type macrophage polarization by activating the JAK2/STAT3 signaling in gastric cancer cells [26]. We questioned whether PGC-1α deficiency could enhance M2 macrophage polarization by modulating the IL-6/STAT3 signaling in I/R livers. To address it, wild-type and PGC-1α (LKO) mice were induced for hepatic I/R injury for 2 weeks and their liver IL-6, STAT3, CD204, fizz1, Ym-1, Collagen I and α-SMA expression were analyzed by immunohistochemistry and Western blot. Compared with the wild-type mice, significantly higher levels of IL-6, STAT3, CD204, fizz1, Ym-1, Collagen I and α-SMA expression were detected in the liver of I/R mice, particularly in the I/R LKO mice, accompanied by severer liver injury and more collagens stained by Sirius red (Fig. 6). Furthermore, TSA treatment dramatically mitigated or abrogated the I/R-induced IL-6, STAT3, Ym-1, CD204 and fizz-1 protein expression detected by immunohistochemistry and Western blot analysis of liver tissues in the mice at 2 and 4 weeks post I/R induction (Supporting Fig. 1A, B). Moreover, RT-qPCR revealed that compared with the Sham group, the I/R group of mice presented significantly higher levels of hepatic IL-10, TGF-β, Arg-1 and Ym-1 mRNA transcripts, which were demolished in the mice with TSA treatment at 2 and 4 weeks post I/R induction (Supporting Fig. 1C). Together, these data evinced that the down-regulated PGC-1α expression by I/R was associated with hepatic macrophage polarization toward M2-type and liver fibrosis in mice by enhancing the IL-6/STAT3 signaling.

### Altered PGC-1α expression modulates the H_2_O_2_-up-regulated IL-6 expression in hepatocytes and M2 polarization of co-cultured macrophages in vitro

**Fig. 7:**
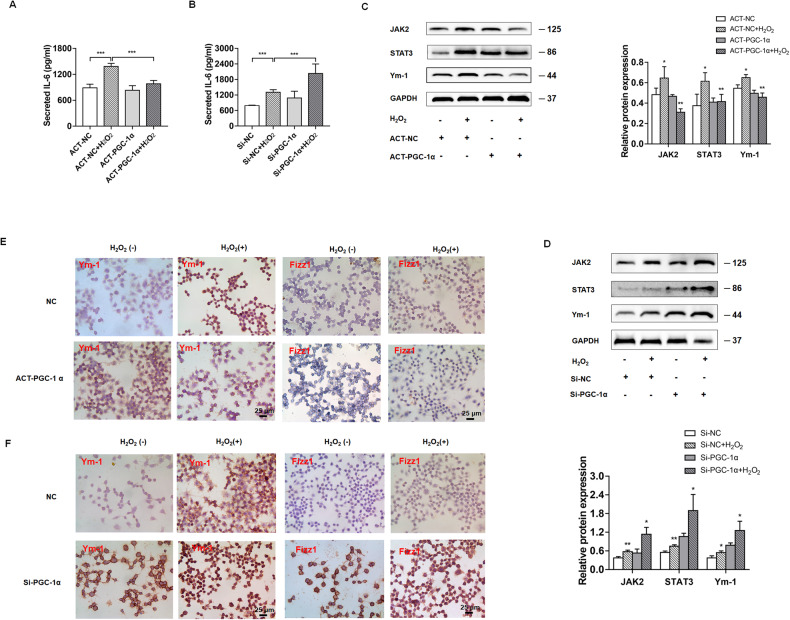
Altered PGC-1α modulates the oxidative stress-stimulated IL-6 production in hepatocytes and the M2 polarization of co-cultured RAW264.7 macrophages in vitro. L-02 cells were transfected with PGC-1α activation plasmid (**A**, **C**, **E**) or PGC-1α siRNA (**B**, **D**, **F**), or control, and treated with 300 μM H_2_O_2_ (+) or untreated (−) for 48 h. **A**, **B** The levels of IL-6 in the supernatants of cultured hepatocytes were assessed by ELISA. **C**, **D** Western blot analysis of the relative levels of JAK2, STAT3, Ym-1 to GAPDH protein expression in RAW264.7 macrophages after co-cultured with the different groups of L-02 cells in transwell plates for 18 h. **E**, **F** Immunocytochemistry analysis of Ym-1, Fizz1 protein expression in RAW264.7 macrophages after co-cultured with the different groups of L-02 cells in transwell plates for 18 h. Data represent 4 independent treatments, *P < 0.05, **P < 0.01, ***P < 0.001.

To test question about whether IL-6 from hepatocytes could promote the M2 polarization of macrophages, L-02 cells were transfected with the plasmid for PGC-1α overexpression or PGC-1α-specific siRNA for PGC-1α silencing in the top inserts of transwell plates in the presence or absence of 300 µM H_2_O_2_ for 48 h. The hepatocytes were cultured with the bottom wells of RAW264.7 macrophages, independent of direct contact for 18 h. Analysis of IL-6 in the supernatants of cultured hepatocytes indicated that PGC-1α overexpression dramatically mitigated the H_2_O_2-_stimulated IL-6 production in hepatocytes (Fig. 7A). Conversely, PGC-1α silencing significantly enhanced the H_2_O_2-_stimulated IL-6 production in hepatocytes (Fig. 7B).

Western blot revealed that the relative levels of JAK2, STAT3 and Ym-1 protein expression in macrophages that had been co-cultured with the H_2_O_2-_treated hepatocytes were obviously higher than that in the macrophages co-cultured with the control hepatocytes without H_2_O_2-_treatment (Fig. 7C). In contrast, following co-cultured with the PGC-1α overexpressing hepatocytes, the macrophages displayed reduced levels of JAK2, STAT3 and Ym-1 protein expression regardless of H_2_O_2-_treatment. Conversely, PGC-1α silencing in hepatocytes clearly enhanced the H_2_O_2-_stimulated JAK2, STAT3 and Ym-1 protein expression in the co-cultured macrophages (Fig. 7D). Similar patterns of Ym-1 and Fizz1 expression were observed by immunocytochemistry in the macrophages after co-cultured with the different groups of hepatocytes (Fig. 7E, F). Collectively, PGC-1α inhibited the H_2_O_2-_stimulated IL-6 production in hepatocytes and limited the M2 polarization of co-cultured macrophages by attenuating the IL-6/JAK2/STAT3 signaling in macrophages.

### Administration of PGC-1α attenuates liver fibrosis post I/R by inhibiting M2-type macrophage polarization

**Fig. 8:**
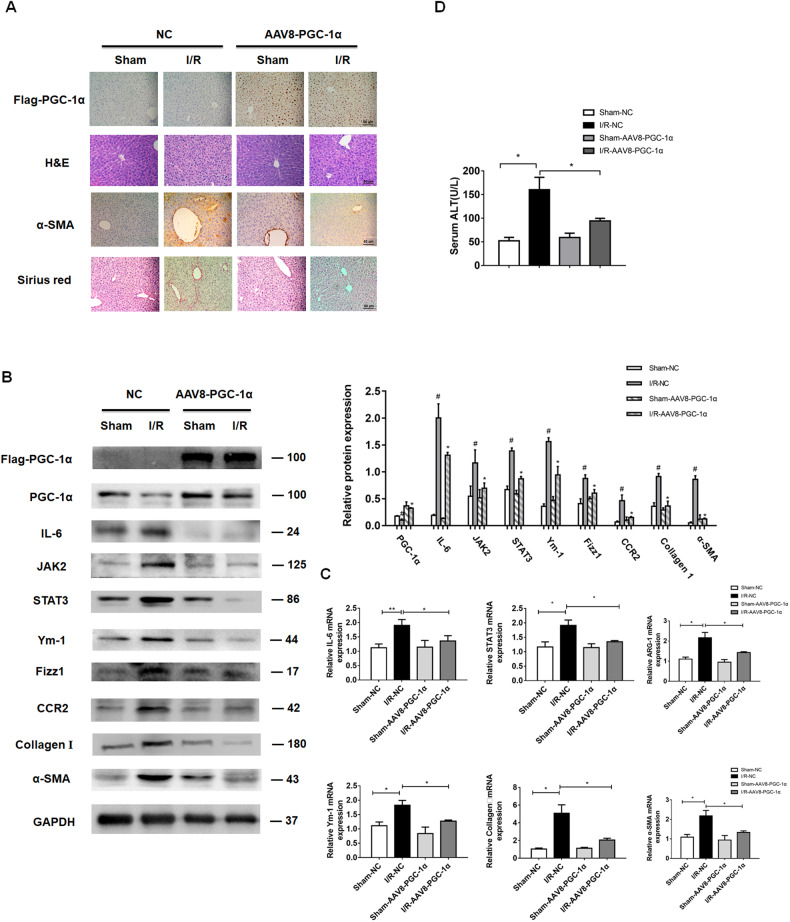
PGC-1α over-expression attenuates the IL-6/STAT3 signaling, M2-type macrophage polarization and liver fibrosis in mice. Three days post hepatic I/R, the mice were randomized and injected with the control AAV8 or AAV8-PGC-1α. Their liver tissues were harvested at 4 weeks post hepatic I/R. **A** H&E, Immunohistochemistry staining of Flag, α-SMA and Sirius red staining. Scale bars: 50 μm. **B** Western blot analyses of Flag, PGC-1α, IL-6, JAK2, STAT3, Ym-1, Fizz1, CCR2, collagen I and α-SMA expression, ^#^*P* < 0.05 vs. the Sham-NC, *P < 0.05 vs. the I/R-NC. **C** RT-qPCR analyses of IL-6, STAT3, Ym-1, Arg-1, collagen I and α-SMA to GAPDH mRNA transcripts. *P < 0.05, **P < 0.01. **D** The levels of serum ALT, *P < 0.05. Data are representative images or shown as mean ± SD of each group (6–8/group) from at least three separate experiments.

Next, we questioned whether administration of exogenous PGC-1α could inhibit M2-type macrophage polarization to attenuate liver fibrosis in mice following I/R induction. To test this hypothesis, 3 days after I/R induction, the mice were randomized and injected intravenously with control AAV8 or AAV8-PGC-1α to induce PGC-1α over-expression. As expected, Western blot and immunohistochemistry demonstrated high levels of PGC-1α expression in the liver of mice receiving AAV8-PGC-1α than those with the control AAV8 at 4 weeks post I/R (Fig. 8A, B). Compared with the control mice, administration with AAV8-PGC-1α obviously decreased liver injury, fibrosis and serum ALT levels in the I/R mice (Fig. 8A, B, D). Furthermore, administration with AAV8-PGC-1α also reduced the relative levels of IL-6, JAK2, STAT3, Ym-1, Arg-1, Fizz1, CCR2, Collagen I and α-SMA expression in the liver of I/R mice at 4 weeks post I/R (Fig. 8B, C). These results further indicated that PGC-1α inhibited M2-type macrophage polarization and attenuated the I/R-induced liver injury and fibrosis, possibly by mitigating the IL-6/ JAK2/STAT3 signaling during the process of I/R injury and fibrosis in mice.

## Discussion

Liver fibrosis is referred to the pathophysiological process of excessive hepatic ECM deposition induced by various pathogenic factors. The dysregulated chronic liver injury and subsequent repair and healing will progress into liver fibrosis, even into cirrhosis and liver failure. At present, liver fibrosis is considered to be a reversible pathological process, and the final result of fibrosis is determined by the balance between the synthesis and degradation of collagen fibers. Activated HSCs can produce much ECM and inhibition of HSC activation can effectively reverse the process of fibrosis [4, 5, 27, 28]. Currently, there is no effectively therapeutic strategy for the inhibition of liver fibrosis.

Hemorrhagic shock, hepatectomy and post-transplantation can cause hepatic IRI, which is one of the most important risk factors associated with the development of liver fibrosis [1, 29]. Hepatic IR can cause oxidative stress and the production of ROS, which can directly damage hepatocytes and induce inflammation, deteriorating liver injury [30]. The role and mechanisms underlying macrophage polarization in liver fibrosis are complicated. While some studies have shown that the numbers of both M1 and M2 macrophages significantly increase in liver fibrosis formation phase [31, 32], other studies have revealed that M2 macrophages can aggravate liver fibrosis [33, 34]. Because both macrophage polarization and the complexity of liver fibrosis, the precise mechanisms by which factors regulate M2-type macrophage polarization during the process of liver fibrosis need to be further clarified.

Naïve macrophages can be classically activated to be M1-type or alternatively into M2-type macrophages with distinct functions. LPS through its receptor of toll-like receptor (TLR)4 can induce M1-type macrophage polarization to produce pro-inflammatory factors to defend against viral infection. IL-4 can promote M2-type macrophage polarization to secrete anti-inflammatory IL-10, IL-4, IL-13 and TGF-β1, which inhibit inflammation, but promote tissue injury repair. In addition, developing M1-type and M2-type macrophages can transdifferentiate into the opposite phenotype under some conditions and the dynamic balance of M1-type and M2-type macrophage functions contributes to environmental inflammatory homeostasis [35–37]. M1/M2 macrophages can be distinguished by the expression of specific marker genes or proteins. These markers include receptors for a variety of transmembrane glycoproteins, scavenger receptors, enzymes, growth factors, hormones, cytokines, and chemokines. In the current study, we found that down-regulated PGC-1α expression increased IL-6 levels in hepatocytes, which was mitigated or abrogated by PGC-1α over-expression. Furthermore, hepatic I/R also increased IL-10, TGF-β, Arg-1, Ym-1, CD204, CCR2 and Fizz-1 expression in the liver of mice at 2 and 4 weeks post I/R. These data indicated that hepatic I/R promoted M2-type macrophage polarization, contributing to the pathogenesis of I/R-related liver fibrosis in mice. It is possible that M2 macrophages may secrete TGF-β to activate HSCs and promote their differentiation into myofibroblasts, leading to the production of ECM and liver fibrosis [35, 38].

PGC-1α can regulate transcription of the related target genes by forming a transcriptional complex. PGC-1α has potent antioxidant and anti-inflammatory activity in mammalian cells. Our previous study has shown that increased ROS inhibits PGC-1α transcription by hampering histone H3 acetylation in the PGC-1α promoter in a rodent model of CCl_4_-induced fibrotic liver, which can be mitigated by TSA [19]. In the current study, we found that the hepatic I/R decreased PGC-1α expression, contributing to liver injury and fibrosis in mice. Furthermore, TSA treatment preserved PGC-1α expression to mitigate the I/R-related liver injury and fibrosis in mice by inhibiting M2-type macrophage polarization (Fig. 4 and Supporting Fig. 1). Similarly, administration of AAV8-PGC-1α to induce systemic PGC-1α over-expression also mitigated the I/R-related M2-type macrophage polarization and liver inflammation, injury and fibrosis in mice (Fig. 8). These results suggest that PGC-1α may a new therapeutic target for the intervention of liver fibrosis by alleviating M2-type macrophage polarization.

The IL-6/STAT3 signaling is important for liver fibrogenesis by promoting HSC activation [23]. In the current studies, we found that down-regulated PGC-1α expression was correlated with increased IL-6 expression in primarily cultured mouse hepatocytes and in the liver of I/R mice. Similarly, treatment with H_2_O_2_ also down-regulated PGC-1α expression, but up-regulated IL-6 expression in cultured hepatocytes. Mechanistically, PGC-1α directly interacted with phosphorylated NF-kBp65 in liver tissues of I/R mice (Fig. 5). Given that activated NF-kBp65 induces IL-6 transcription [25, 39] the down-regulated PGC-1α expression by hepatic I/R would allow more free phosphorylated NF-kBp65 to induce IL-6 transcription in the liver of I/R mice. Subsequently, the increased IL-6 enhanced the JAK/SATA3 signaling to promote M2-type macrophage polarization, which secreted TGF-β and activated HSCs, leading to excessive ECM production and deposition in the liver, contributing to the pathogenesis of fibrosis in mice. Administration of AAV8-PGC-1α or TSA to increase PGC-1α mitigated the I/R-promoted M2-type macrophage polarization by inhibiting the IL-6/STAT3 signaling, and alleviating liver fibrosis in I/R mice. These results suggest that PGC-1α may be a critical regulator of hepatic macrophage polarization and liver fibrosis.

In conclusion, the current study found that PGC-1α deficiency deteriorated the I/R-induced liver fibrosis by enhancing the IL-6/JAK2/STAT3 signaling and M2-type macrophage polarization. Preserving and enhancing PGC-1α expression and activity may be new potential therapeutic strategies for the treatment of liver fibrosis.

## Materials and Methods

### Reagents

Special reagents included antibodies against Collagen I, alpha smooth muscle actin (α-SMA), GAPDH, TNF-α, IL-6, JAK2, STAT3 (Cell Signaling Technology, Beverly, MA, USA); Ym-1 (Abcam, Cambridge, MA, USA), PGC-1α, phospho-NF-kBp65, NF-kBp65, CD204, resistin-like molecule alpha 1 (Fizz1), Flag, CCR2, nitrotyrosine, lipofectamine 2000 and Trizol (Invitrogen, Carlsbad, CA, USA), TSA (Sigma-Aldrich, St Louis, MO, USA), PGC-1α CRISPR activation plasmids (Santa Cruz Biotechnology, Santa Cruz, CA, USA), PGC-1α-specific small interfering RNAs (siRNAs) and negative control siRNAs (Table 1) (Oligobio, Beijing, China). Other specific reagents contained alanine aminotransferase (ALT) assay kits (Jiancheng Biological Engineering Institute, Nanjing, Jiangsu, China), Sirius red biochemical reagent (Solarbio Life Science, Beijing, China) and Streptomycin-biotin peroxidase immunohistochemical staining kits (Maixin Biological Technology, (Fuzhou, Fujian, China).

**Table 1:** The sequences of RNA and DNA oligonucleotides.

Name	Sense Strand/Sense Primer (5’-3’)	Sense Strand/Sense Primer (5’-3’)
**siRNA duplexes**		
siPGC-1α	GCAAUAAAGCGAAGAGUAUTT	AUACUCUUCGCUUUAUUGCTT
siPGC-1α	CCACCACUCCUCCUCAUAATT	UUAUGAGGAGGAGUGGUGGTT
siPGC-1α	GCUACCUGAGAGAGACUUUTT	AAAGUCUCUCUCAGGUAGCTT
NC	UUCUCCGAACGUGUCACGUTT	ACGUGACACGUUCGGAGAATT
**Primers for qPCR**		
PGC-1α (mouse)	TATGGAGTGACATAGAGTGTGCT	GTCGCTACACCACTTCAATCC
PGC-1α (human)	TGGTGCCACCATCAAAGA	TCACCAAACAGCCGCAGACTG
Sod1 (mouse)	GCCCGGCGGATGAAGA	CGTCCTTTCCAGCAGTCACA
Sod3 (mouse)	CAGACAAAGGAGCGCAAGAAG	TGAGGCTTAAGTGGTCTTGCA
Mrc-1 (mouse)	GGGACGTTTCGGTGGACTGTGG	TTGTGGGCTCTGGTGGGCGA
Arg1 (mouse)	GATGTCCCTAATGACAGCTCC	AGCACCACACTGACTCTTCC
Ym-1 (mouse)	CCCCAGGAAGTACCCTATGCCT	AACCACTGAAGTCATCCATGTCC
Collagen I (mouse)	GCT CCT CTT AGG GGC CAC T	CCA CGT CTC ACC ATT GGG G
α-SMA (mouse)	GTCCCAGACATCAGGGAGTAA	TCGGATACTTCAGCGTCAGGA
TGF-β (mouse)	CTCCCGTGGCTTCTAGTGC	GCCTTAGTTTGGACAGGATCTG
TNF-α (mouse)	CATCTTCTCAAAATTCGAGTGACAA	TGGGAGTAGACAAGGTACAACCC
IL-1β (mouse)	CCGTGGACCTTCCAGGATGA	GGGAACGTCACACACCAGCA
IL6 (mouse)	AGTTGCCTTCTTGGGACTGA	TCCACGATTTCCCAGAGAAC
MCP1 (mouse)	TAAAAACCTGGATCGGAACCAAA	GCATTAGCTTCAGATTTACGGGT
IL-10 (mouse)	CCAAGCCTTATCGGAAATGA	TTTTCACAGGGGAGAAATCG
HGF (mouse)	AAAGGGACGGTATCCATCACT	GCGATAGCTCGAAGGCAAAAAG
STAT3 (mouse)	CACCTTGGATTGAGAGTCAAGAC	AGGAATCGGCTATATTGCTGGT
GAPDH (mouse)	ACCACAGTCCATGCCATCAC	ACGGATACATTGGGGGTAGG
Nrf2 (mouse)	GGTTGCCCACATTCCCAAAC	TCCTGCCAAACTTGCTCCAT
GAPDH (human)	GAGTCAACGGATTTGGTCGT	GACAAGCTTCCCGTTCTCAG

*NC* Negative control.

### A mouse model of ischemia-reperfusion (I/R) injury and treatments

Male wild-type (WT) C57BL/6 J mice at 8 weeks of age were obtained from Beijing Vital River Laboratory Animal Technology, China. Ppargc1α^f/f^ (B6.Cg-Ppargc1α^tm2.1Brsp^/J) and Tg (Alb-cre)^21Mgn^/J (Alb-cre^+/+^) mice were obtained from the Jackson Laboratory (Bar Harbor, ME, USA). Hepatocyte-specific PGC-1α knockout (Ppargc1α^f/f^ Alb-cre^+/-^, LKO) mice were generated in our animal research facility using the classic Cre-loxP recombination system [19, 40]. All mice were housed in a specific pathogen-free environment at 22 ± 2 °C, 40 ± 5% relative humidity, and 12 h light/12 h dark cycle. The experimental protocols were approved by the Animal Care and Use Committee of Capital Medical University, and the experiments were performed, according to the guidelines of the National Institutes of Health (NIH).

Before induction of I/R injury, the mice were randomized and treated intraperitoneally with 0.6 mg/kg TSA or vehicle daily for two days. Some mice were subjected to induction of segmental (70%) hepatic I/R injury as described previously [41]. Briefly, individual mice were injected intraperitoneally with 1% sodium pentobarbital (50 mg/kg) and subjected to a midline laparotomy, followed by clamping the vasculature for the left and median 3 lobes (ischemic lobes) of their liver using an atraumatic microvascular clamp for 60 min and reperfusion. The mice in the sham group received the same surgery without vasculature clamping. Accordingly, there were the Sham, Sham-TSA, I/R, and IR-TSA four groups of mice. The operator or investigator was blinded to the grouping. Some mice from each group were euthanized at 1, 3, 6, 12, 24 h, 2 or 4 weeks post reperfusion and their serum and liver tissue samples were collected.

In addition, some mice from each group were treated intravenously with 1.1 × 10^11^ transducing U/100 μl of AAV8-PGC-1α or control AAV8 (Hanbio, Shanghai, China) through the tail vein at 3 days post I/R or Sham operation. At 4 weeks post viral injection, their liver tissues were collected. The levels of serum ALT in individual mice were measured using the specific kit, per the manufacturer’s instruction.

### Histology

The collected liver tissues were fixed in 10% of formalin overnight and paraffin-embedded. The liver tissue sections (4 μm) were routine-stained with hematoxylin and eosin (H&E) or Sirus red.

### Immunohistochemistry and immnocytochemistry

The levels of specific protein expression in the liver of individual mice were analyzed by immunohistochemistry. Similarly, the cells were fixed in 4% paraformaldehyde and analyzed by immnocytochemistry. The liver tissue sections (4 μm) were dewaxed, rehydrated and blocked, followed by probing with anti-α-SMA (1:800), anti-Collagen I (1:800), anti-IL-6 (1:800), anti-STAT3 (1;800), anti-PGC-1α (1:1000), anti-CD204 (1:800), anti-Fizz1(1:1000), anti-Flag (1:800), anti-CCR2 (1:800), anti-nitrotyrosine (1:1000) and anti-Ym-1 (1:1200) overnight at 4 °C. The cell samples were incubated with anti-Fizz1 (1:200) and anti-Ym-1 (1:200) overnight at 4 °C. After being washed, the bound antibodies were reacted with biotinylated secondary antibodies, and subsequently with HRP-conjugated streptavidin. The immune signals were visualized with 3,3′-diaminobenzidine (DAB) and the sections and cells were counterstained with hematoxylin.

### Cell culture

human non-tumor hepatic L-02 cells were obtained from ATCC (Manassas, VA, USA). Mouse primary hepatocytes were isolated from C57BL/6 mice as described previously [19, 40]. L-02 cells and primary hepatocytes were cultured in Dulbecco’s modified Eagle’s medium supplemented with 10% of fetal bovine serum (FBS) at 37 °C in a humidified incubator of 5% CO_2_. The cells were transfected with a PGC-1α (accession code: NM-013261) CRISPR activation plasmid, PGC-1α siRNAs, the control plasmid or siRNA using Lipofectamine 2000, following the manufacturer’s protocols. After transfection for 24 h, the cells were treated with 300 or 100 μM H_2_O_2_ for 48 h and harvested for analysis of cellular PGC-1α and IL-6 expression.

### Enzyme-linked immunosorbent assay (ELISA)

After transfection and treated with 300 μM H_2_O_2_ for 48 h, the supernatants of cultured L-02 cells were harvested for analysis of IL-6 levels using an ELISA kit (Lianke Biological Engineering Institute, Hangzhou, Zhejiang, China), according to the manufacturer’s instructions.

### Transwell co-culture assay of L-02 cells and RAW macrophages

After transfection and treated with 300 μM H_2_O_2_ for 48 h, the L-02 cells were cultured in the top cell culture insert with a pole size of 1.0 µm (Labselect, Cat: 14152) in 24-well transwell and the bottom wells were added with RAW 264.7 macrophages (ATCC TBI-71). The cells were co-cultured for 18 h and the macrophages were collected for characterizing their phenotypes by immunocytochemistry using anti-Ym-1 and anti-Fizz1 antibodies. The relative levels of JAK2, STAT3 and Ym-1 in macrophages were quantified by Western blot.

### Real-time quantitative PCR (RT-qPCR)

Total RNA was extracted from liver tissues, L-02 cells and primary hepatocytes using Trizol reagent following the manufacturer’s protocols and reverse-transcribed into cDNA. The relative levels of target gene mRNA transcripts to the control GAPDH were quantified by RT-qPCR using the SYBR Green Master mix and specific primers (Table 1) on the ABI prism 7500 detection system (Applied Biosystems, Foster City, CA). The data were analyzed by 2^-ΔΔCt^.

### Western blot

Individual liver tissue samples were homogenized and the cell samples were lysed, followed by centrifuged. After quantification of protein concentrations, individual samples (30 µg/lane) were separated by SDS polyacrylamide electrophoresis on 12% gels and transferred onto polyvinylidene fluoride membranes (Merck Millipore, Billerica, MA, USA). The membrane was blocked with 5% BSA in TBST for 1 h at room temperature and incubated at 4 °C overnight with anti-STAT3 (1:2000), anti-JAK2 (1:2000), anti-Ym-1 (1:2000), anti-PGC-1α (1:2000), anti-IL-6 (1:2000), anti-TNFα (1:2000), anti-phospho-NF-kBp65 (1:2000), anti-α-SMA (1:2000), anti-Collagen I (1:2000), anti-Fizz1 (1:2000), anti-Flag (1:2000), anti-CCR2 (1:2000), anti-nitrotyrosine (1:1000) and anti-GAPDH (1:6000). After being washed, the membranes were reacted with HRP-conjugated secondary antibodies and visualized using enhanced chemiluminescence (Bio-Rad; Hercules, CA, USA). The images were analyzed using ImageJ software (NIH).

### Co-immunoprecipitation

The potential interactions of proteins were analyzed by coimmunoprecipitation. The liver tissue homogenates were probed with anti-PGC-1α or a control mouse IgG. After being precipitated with Protein A/G beads, the immunoprecipitated proteins were resolved by SDS-PAGE and analyzed by Western blot using anti-phospho-NF-kBp65 (1:2000).

### Data analysis

Data are expressed as mean ± standard deviation (SD). Comparisons between group were evaluated by two-tailed Student’s t test and the difference among the groups was analyze by ANOVA and post hoc Bonferroni test using the SPSS12.0 software. Statistical significance was defined when a *P*-value of < 0.05.

### Supplementary information

Supplementary figure legends

Supporting Figure 1

Uncropped western blots

## Data Availability

All data are available in the main text or the supplementary materials.
