# Supplementary material for: PGC-1α inhibits M2 macrophage polarization and alleviates liver fibrosis following hepatic ischemia reperfusion injury
Source: Cell Death Discov. 2023 Sep 7;9:337. doi: 10.1038/s41420-023-01636-2 (PMC10484946; doi:10.1038/s41420-023-01636-2)
Supplement: Supplementary file 1 — Supplementary figure legends [file 41420_2023_1636_MOESM1_ESM.docx]

**Supplementary figure legends**

**Supporting Figure 1. TSA treatment preserves PGC-1α expression to mitigate M2-type macrophage polarization by attenuating the IL-6/STAT3 signaling in the liver of I/R mice**

Two and four weeks after hepatic I/R, the levels of IL-6, STAT3, Ym-1, CD204, Fizz1, expression were tested by immunohistochemistry (A), Western blot (B) and RT-qPCR (C). Data are representative images or shown as mean ± SD of each group (4-8/group) from at least four separate experiments. **P*<0.05, **P < 0.01, ***P < 0.001.
