# Supplementary figures and images for: PGC-1α inhibits M2 macrophage polarization and alleviates liver fibrosis following hepatic ischemia reperfusion injury
Source: Cell Death Discov. 2023 Sep 7;9:337. doi: 10.1038/s41420-023-01636-2 (PMC10484946; doi:10.1038/s41420-023-01636-2)

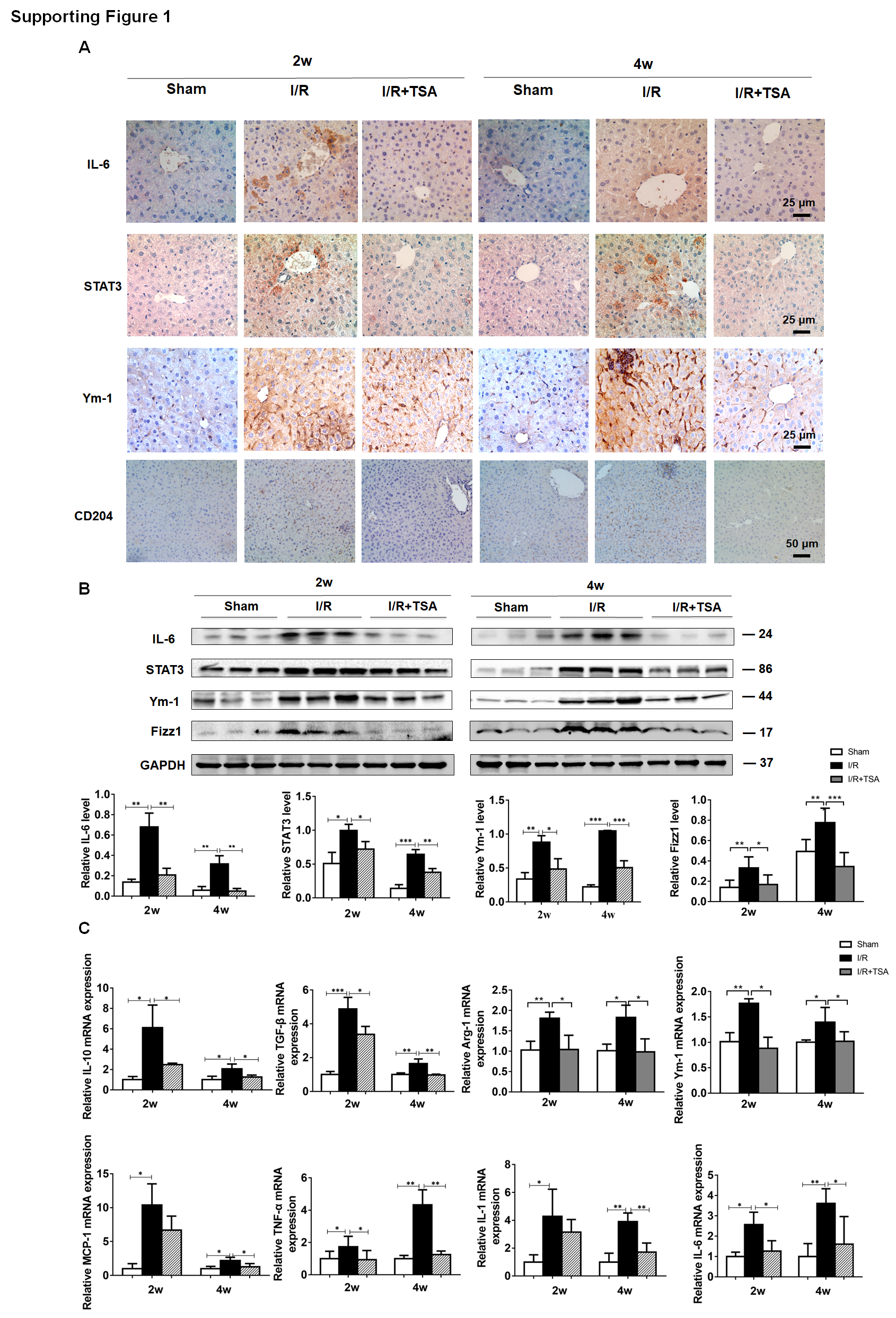

Supplement: Supplementary file 2 — Supporting Figure 1 [file 41420_2023_1636_MOESM2_ESM.tif]

Fig.1

B

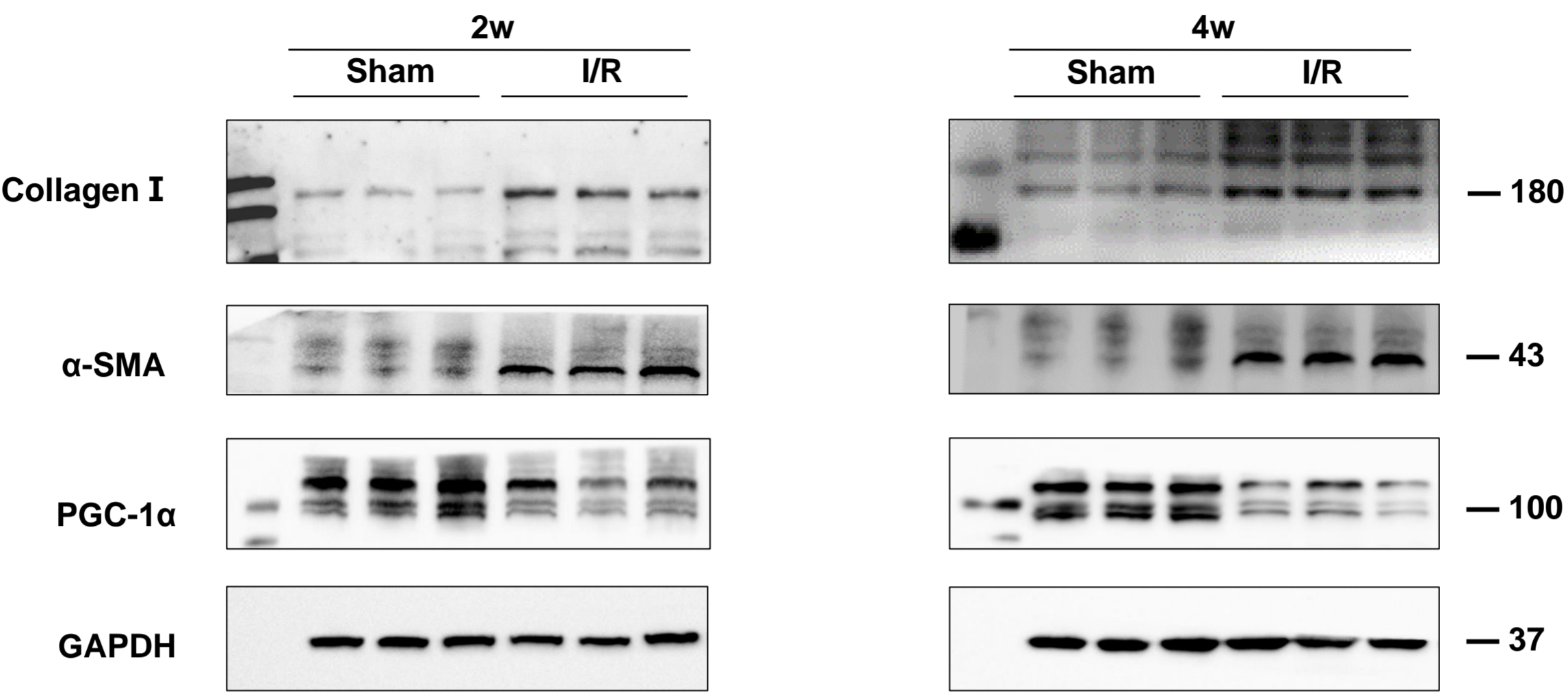

Fig.2

B

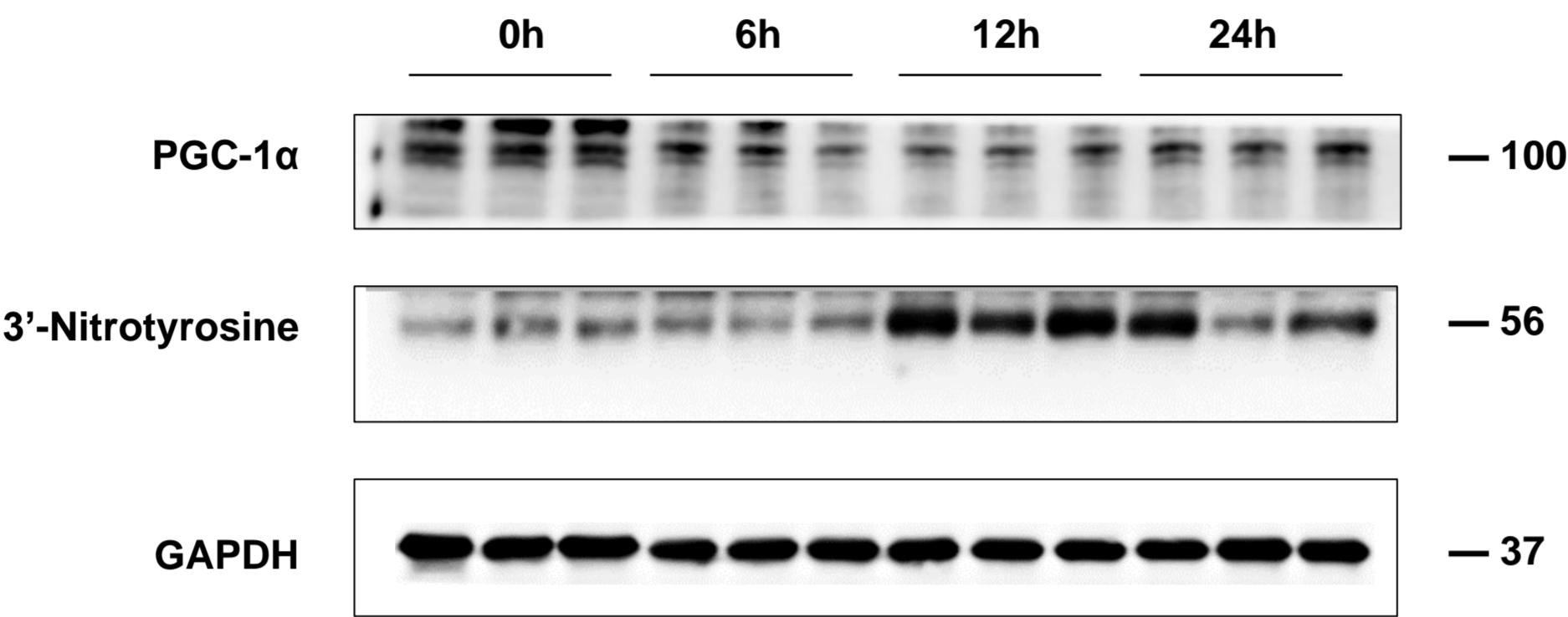

E

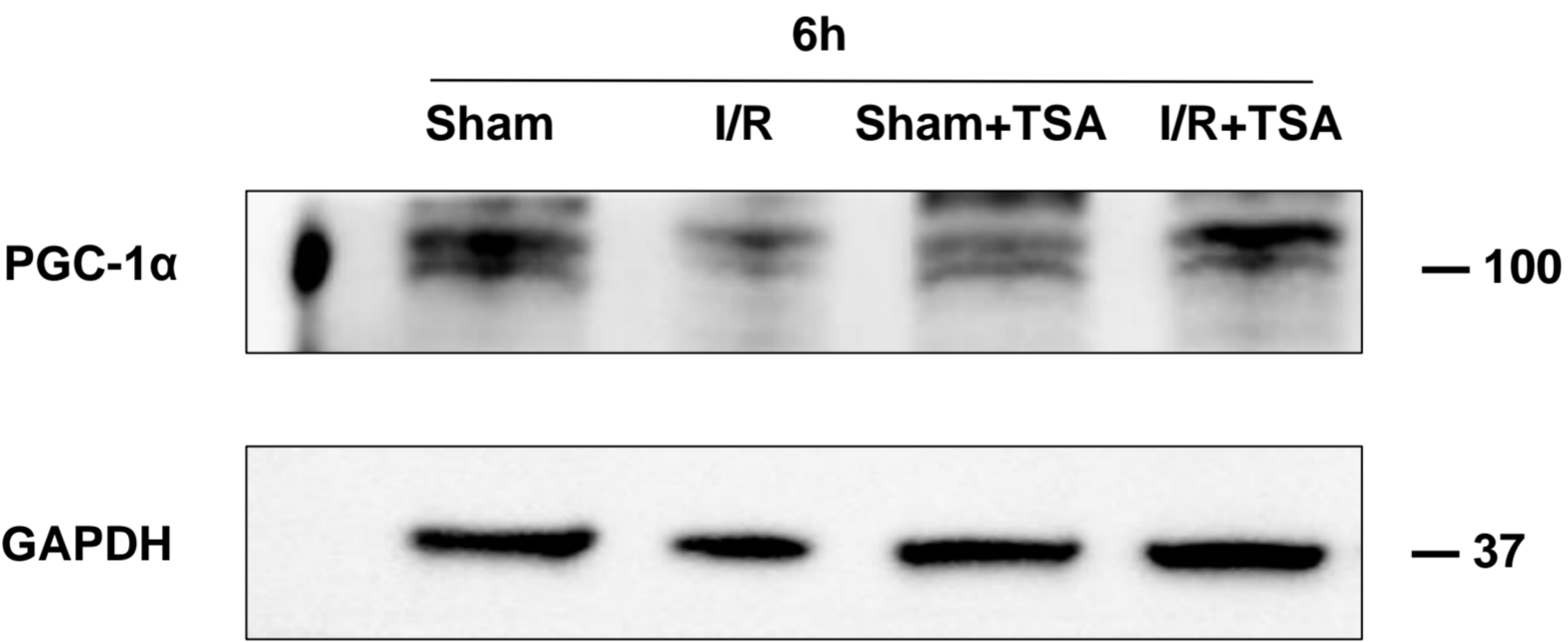

Fig.3

B

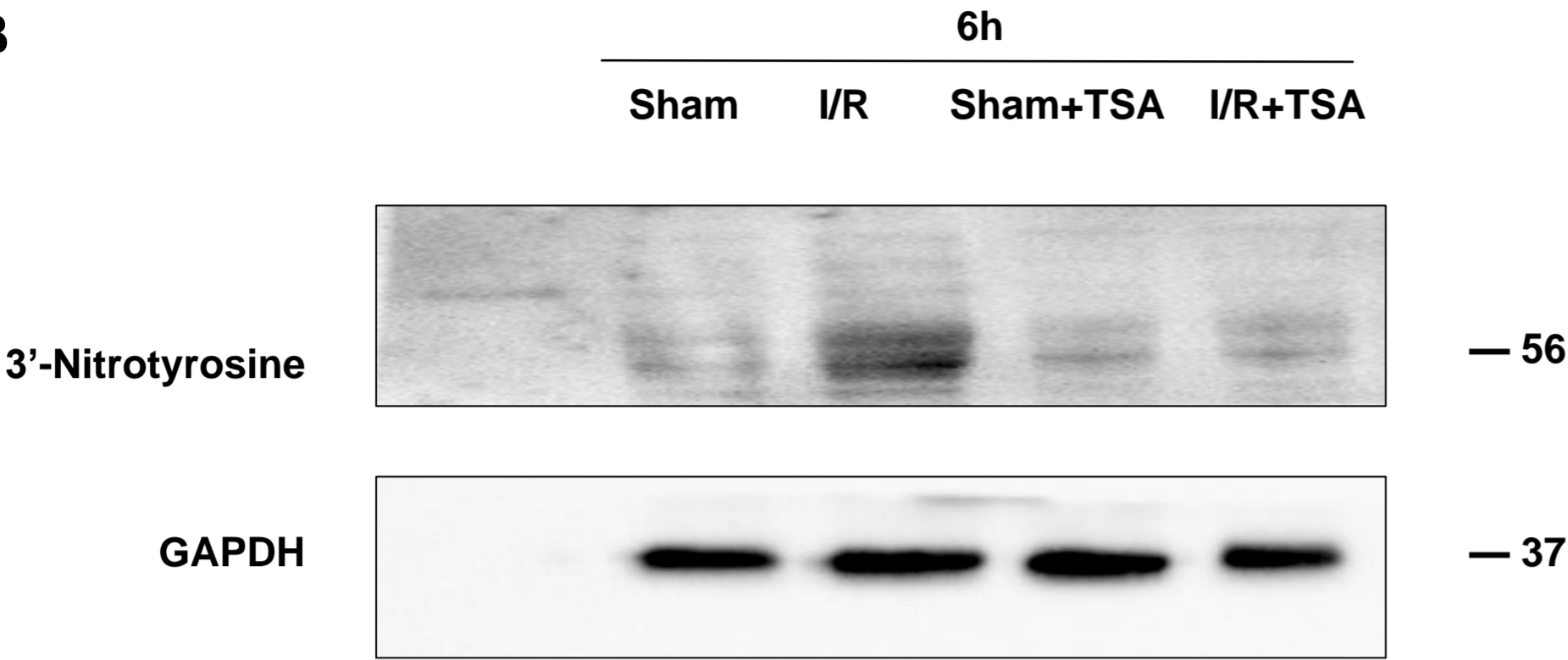

F

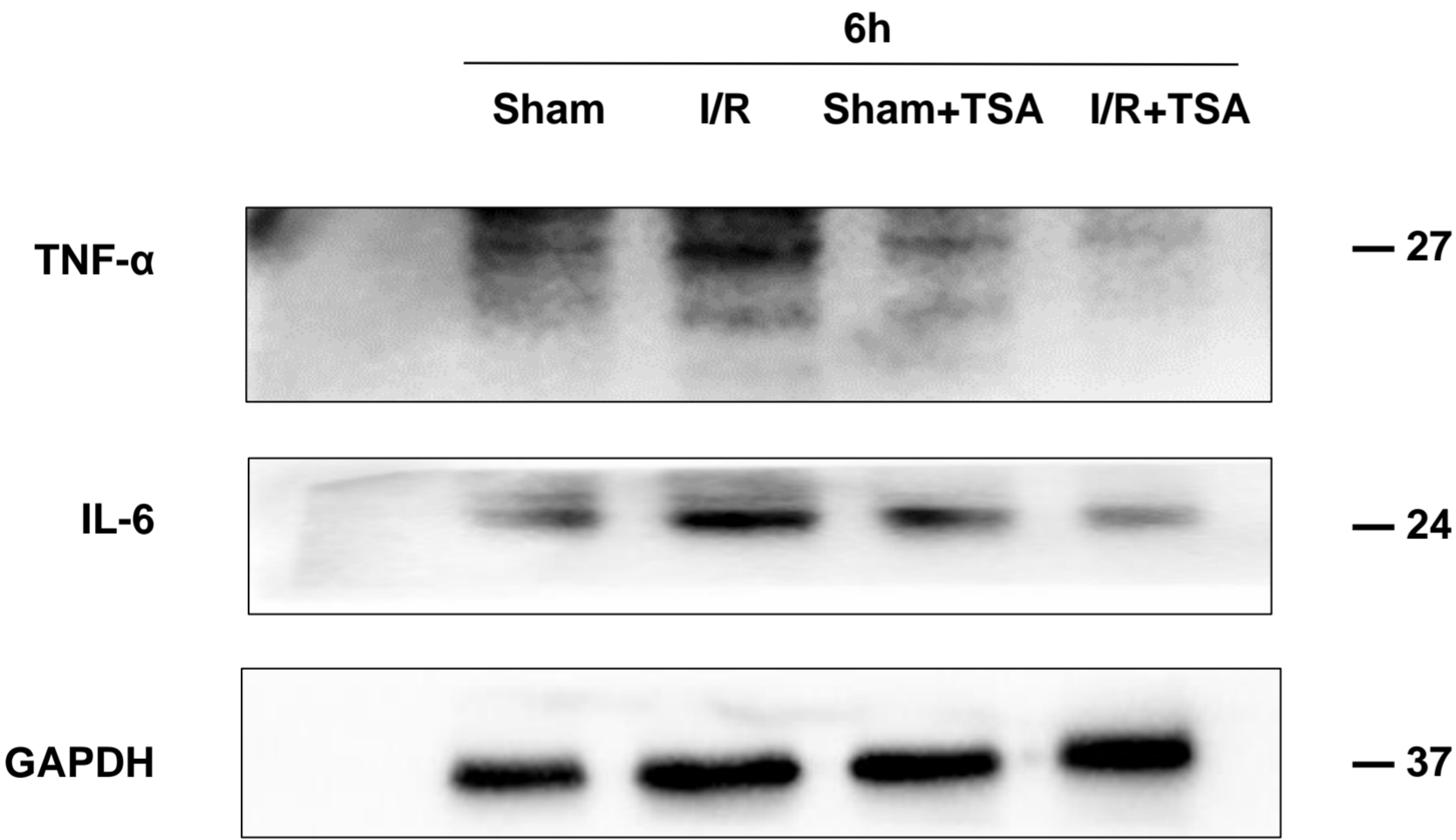

Fig. 4

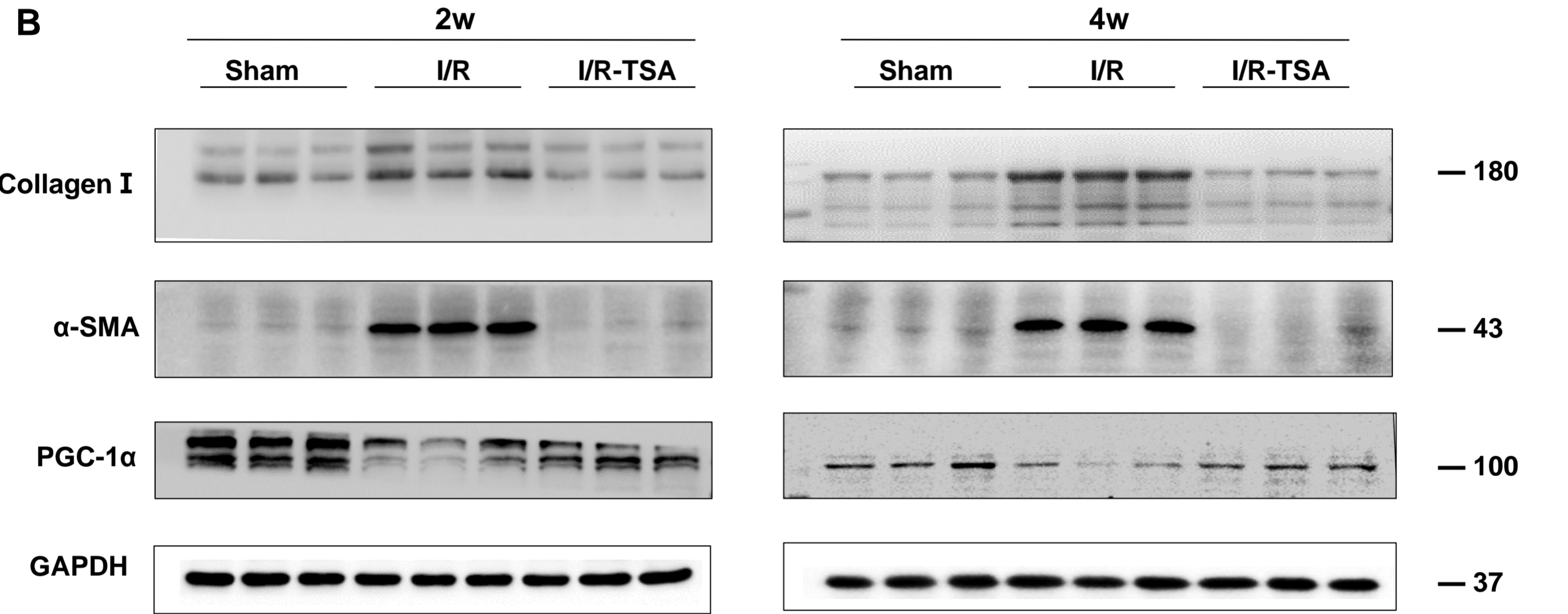

Fig. 5

A

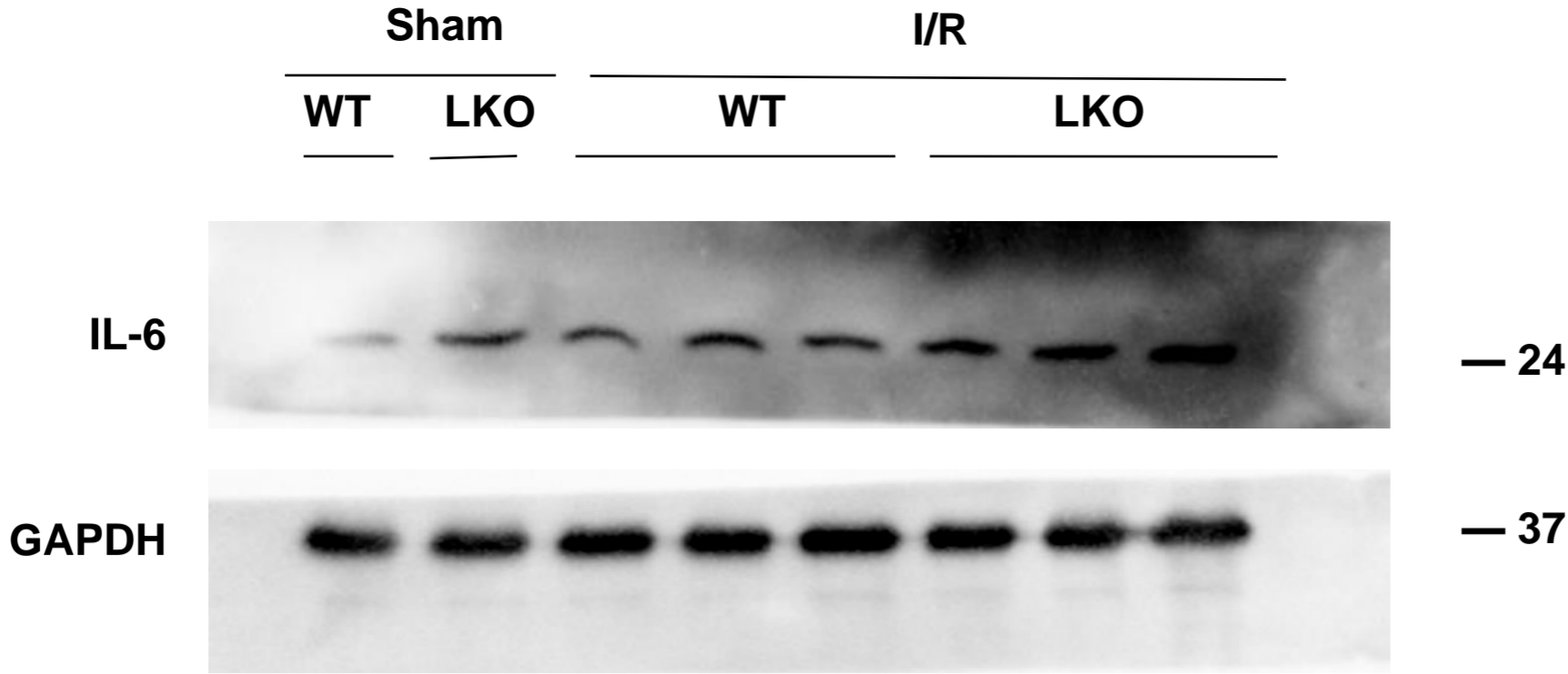

D

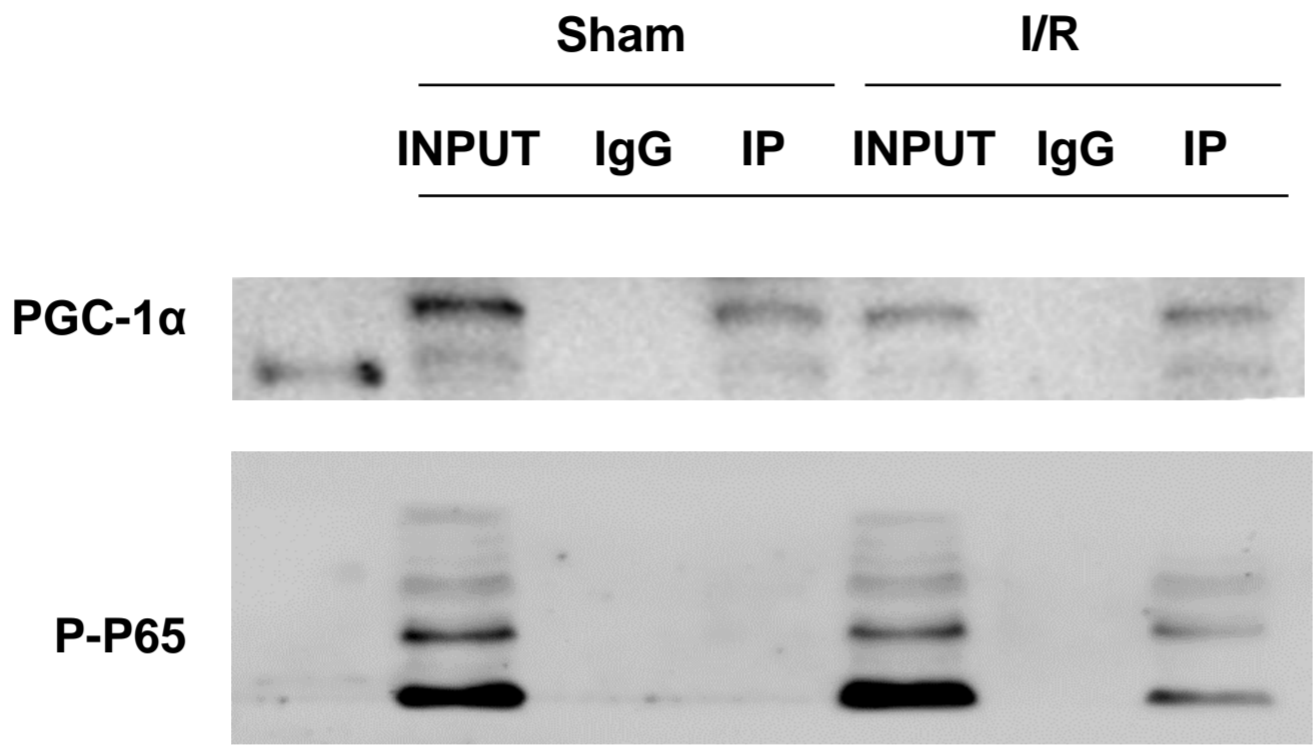

C

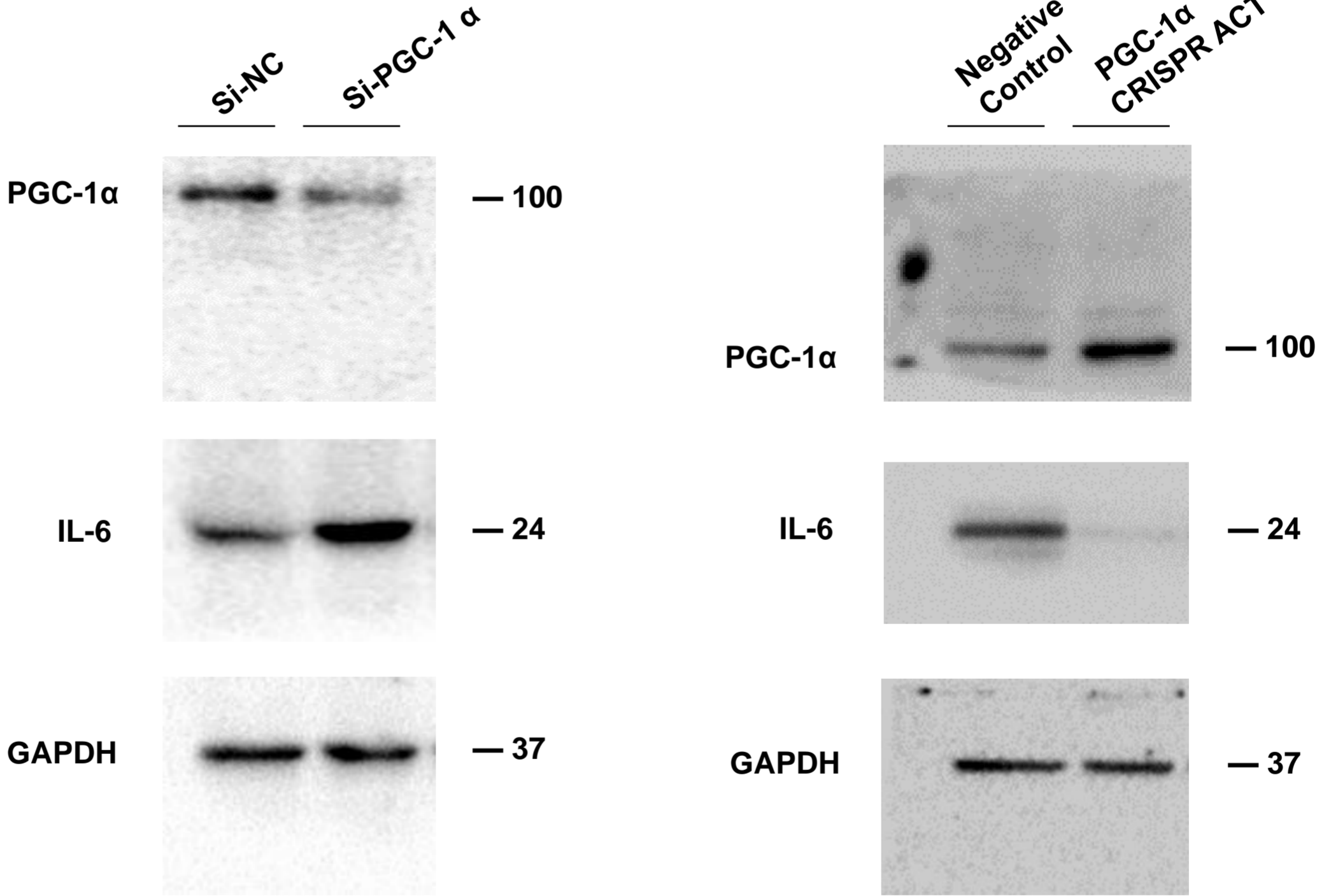

E

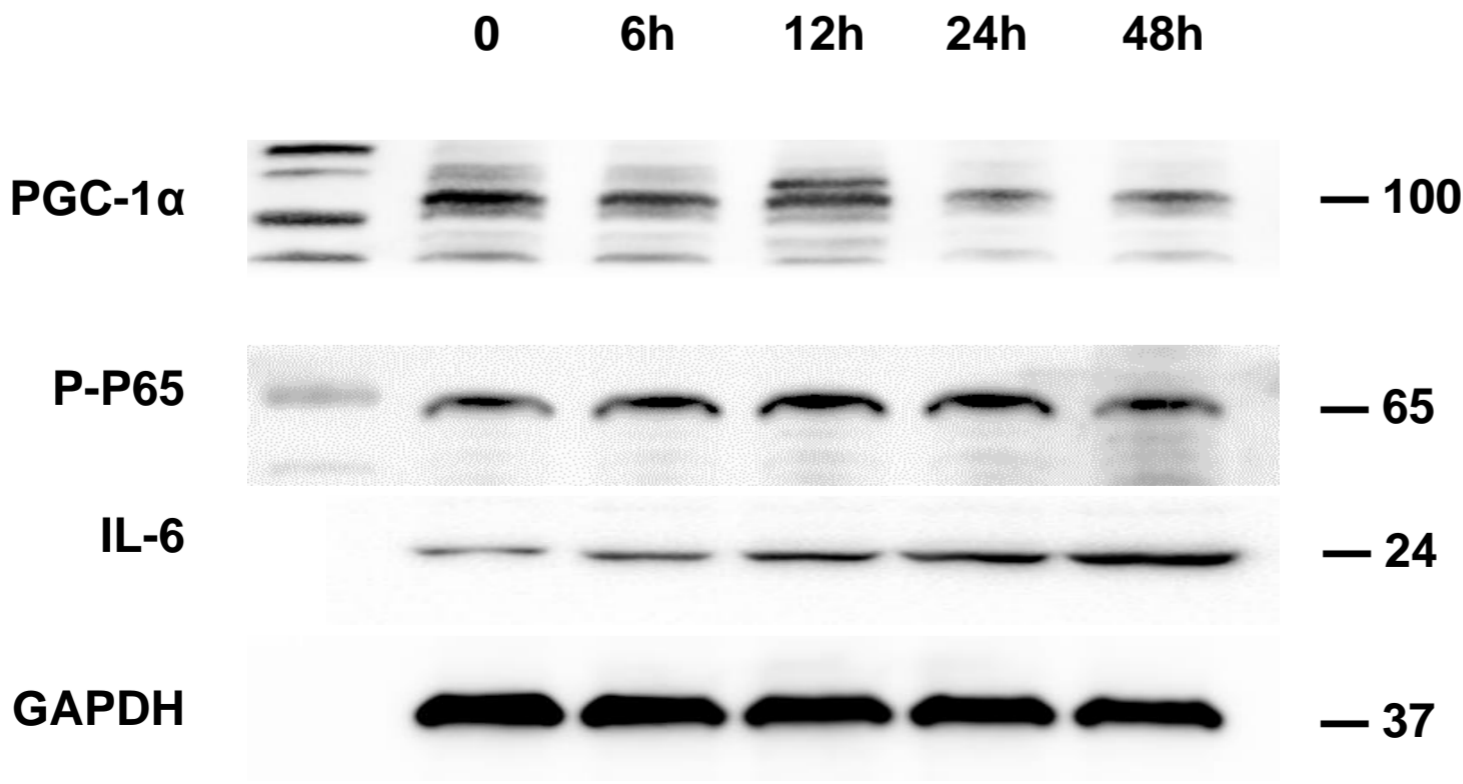

Fig. 6

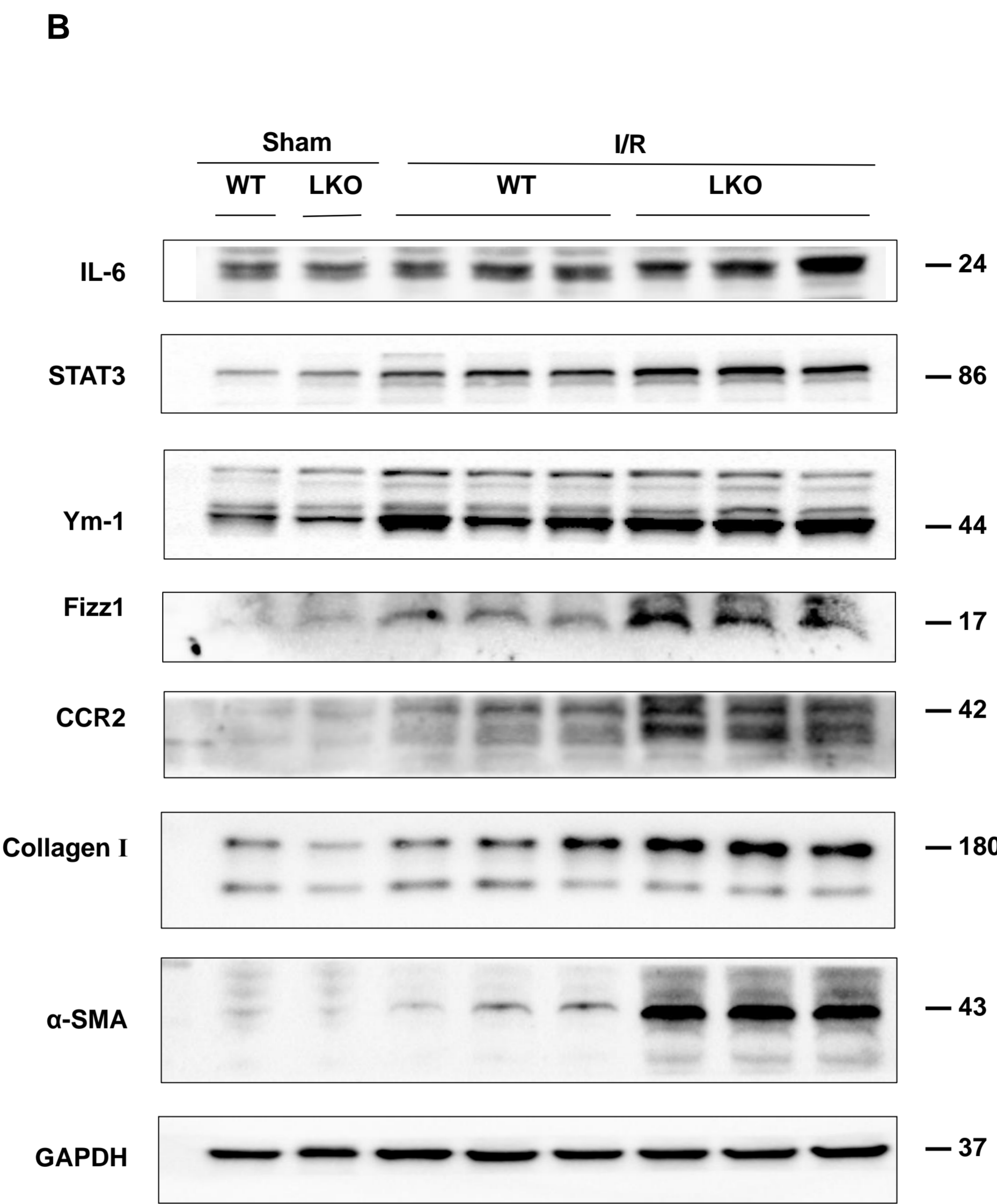

Fig. 7

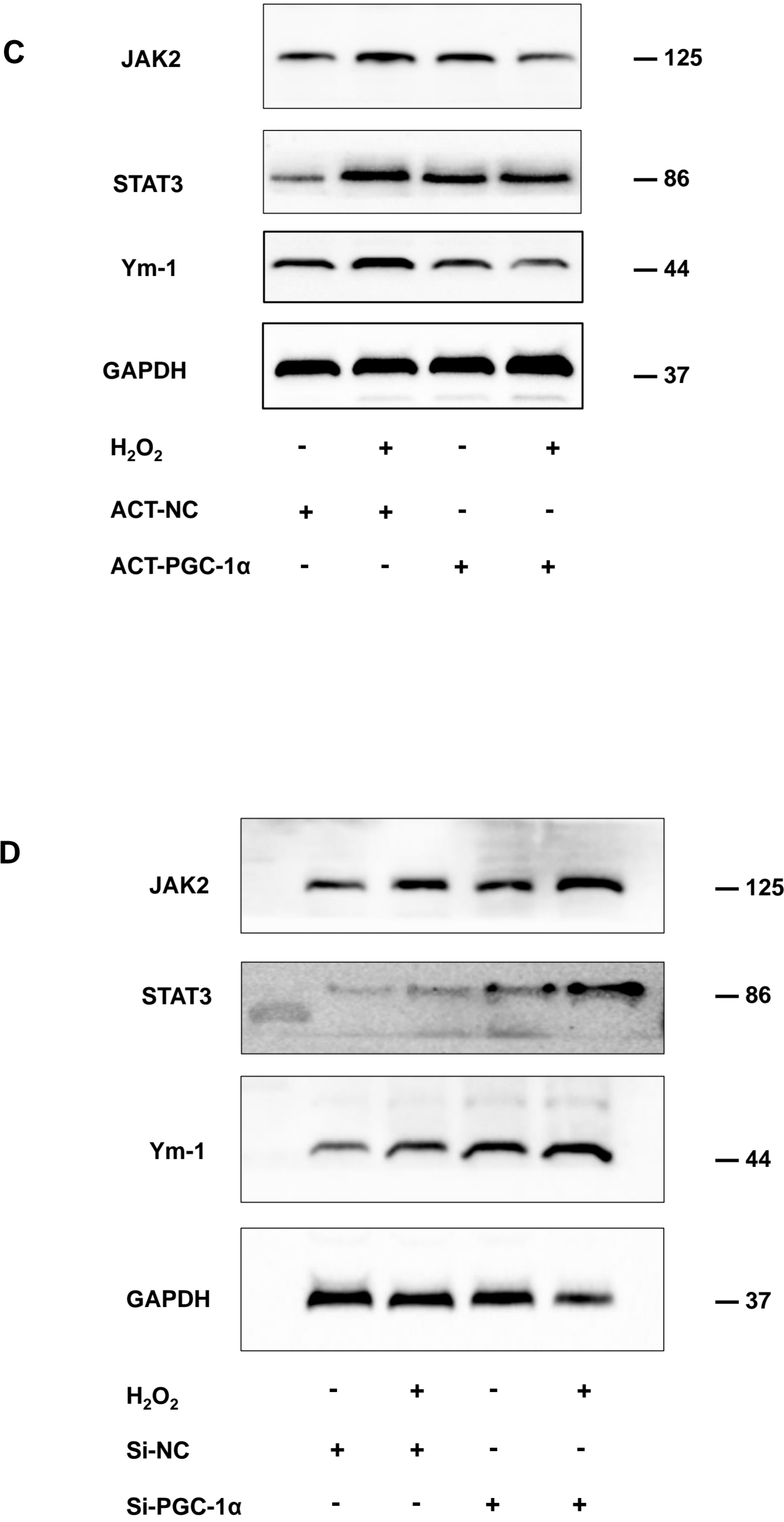

Fig. 8

B

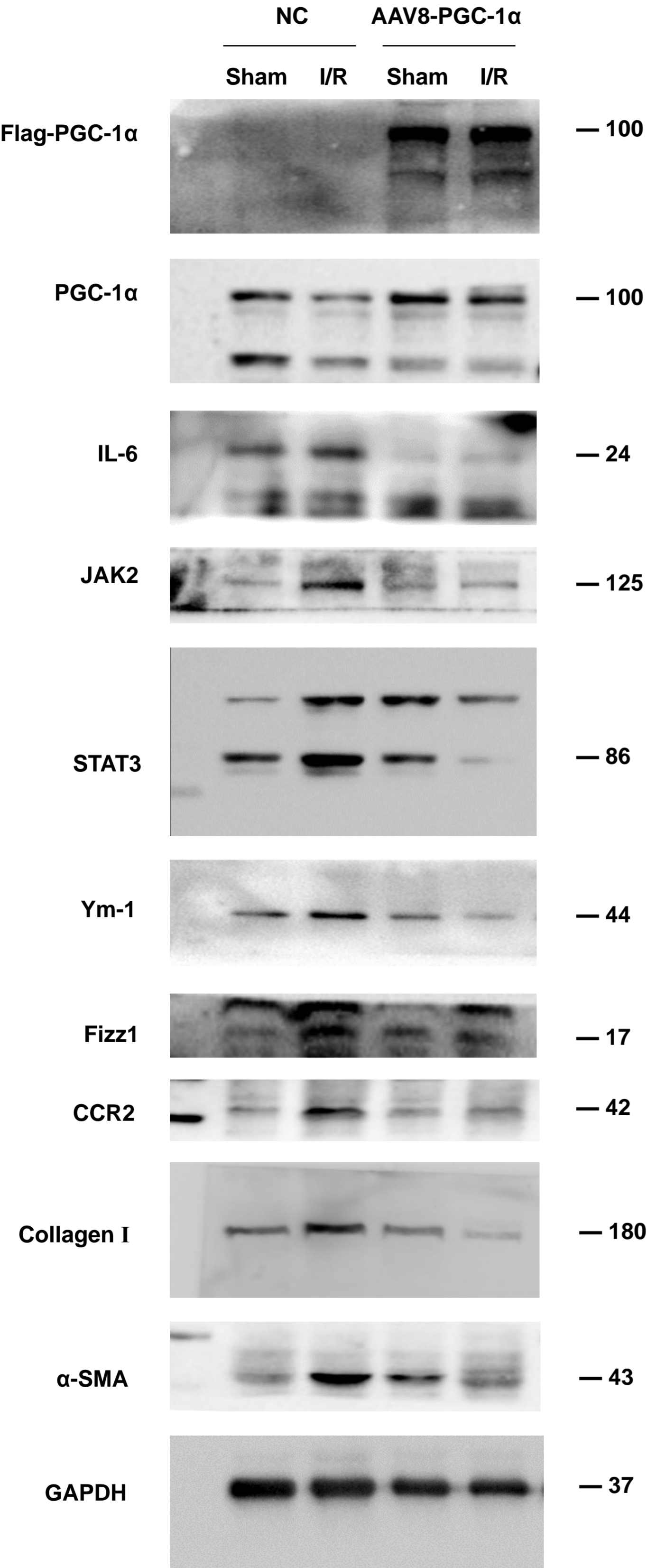

Supporting Figure 1

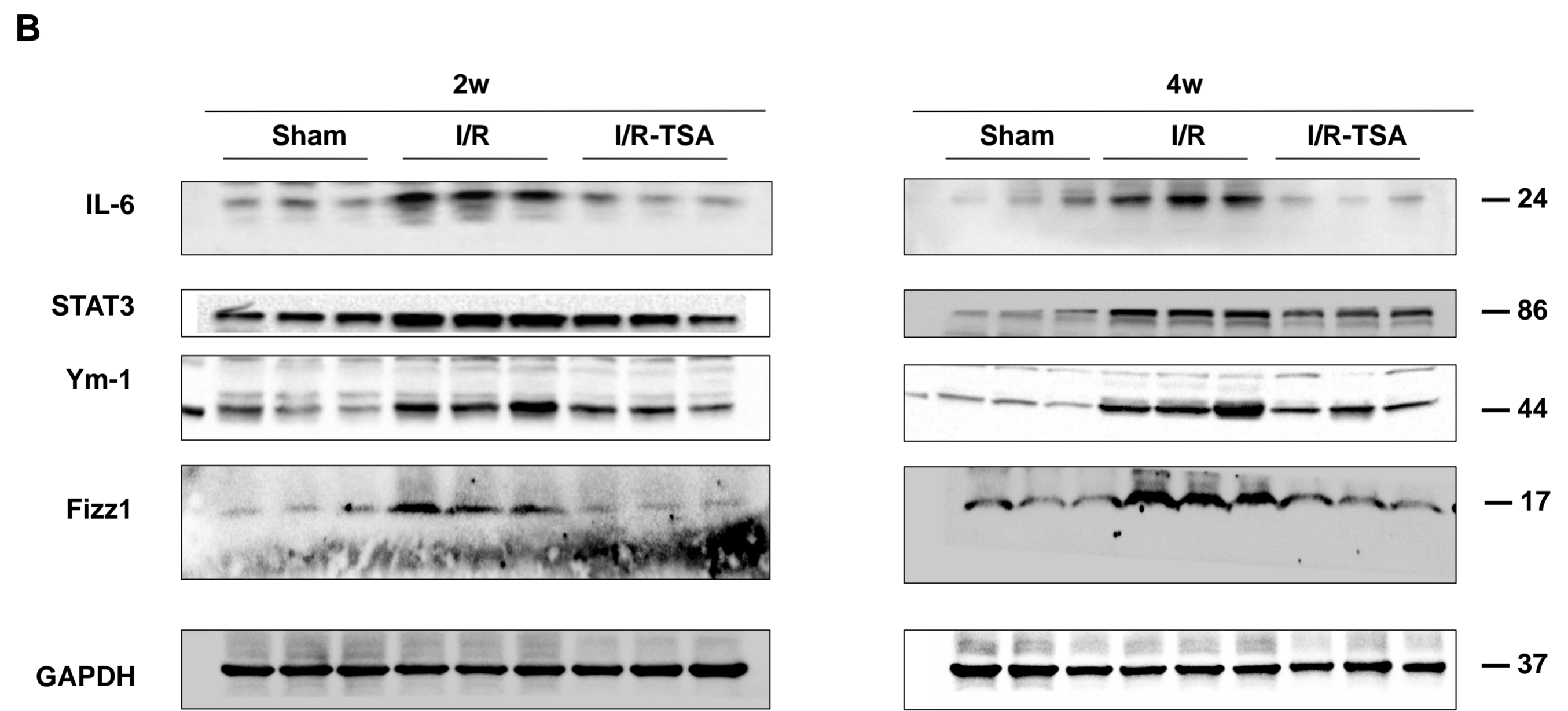

Supplement: Supplementary file 3 — Uncropped western blots [file 41420_2023_1636_MOESM3_ESM.pdf]
